# Sex and gametogenesis stage are strong drivers of gene expression in *Mytilus edulis* exposed to environmentally relevant plasticiser levels and pH 7.7

**DOI:** 10.1007/s11356-022-23801-3

**Published:** 2022-11-02

**Authors:** Luana Fiorella Mincarelli, Emma C. Chapman, Jeanette M. Rotchell, Alexander P. Turner, Katharina C. Wollenberg Valero

**Affiliations:** 1 Department of Biological and Marine Sciences, University of Hull, Hull, HU6 7RX UK; 2 Department of Computer Science, University of Nottingham, Nottingham, NG8 1BB UK

**Keywords:** *M. edulis*, DEHP, pH, Sex differences, Gametogenesis stage, Stress response, Carbonic anhydrase, Oestrogen receptor-like

## Abstract

Plastic pollution and changes in oceanic pH are both pressing environmental issues. Little emphasis, however, has been placed on the influence of sex and gametogenesis stage when investigating the effects of such stressors. Here, we examined histology and molecular biomarkers of blue mussels *Mytilus edulis* exposed for 7 days to a pH 7.7 scenario (− 0.4 units) in combination with environmentally relevant concentrations (0, 0.5 and 50 µg/L) of the endocrine disrupting plasticiser di-2-ethylhexyl phthalate (DEHP). Through a factorial design, we investigated the gametogenesis cycle and sex-related expression of genes involved in pH homeostasis, stress response and oestrogen receptor-like pathways after the exposure to the two environmental stressors. As expected, we found sex-related differences in the proportion of developing, mature and spawning gonads in histological sections. Male gonads also showed higher levels of the acid–base regulator *CA2*, but females had a higher expression of stress response-related genes (i.e. *sod*, *cat*, *hsp70*). We found a significant effect of DEHP on stress response-related gene expression that was dependent on the gametogenesis stage, but there was only a trend towards downregulation of *CA2* in response to pH 7.7. In addition, differences in gene expression between males and females were most pronounced in experimental conditions containing DEHP and/or acidified pH but never the control, indicating that it is important to consider sex and gametogenesis stage when studying the response of mussels to diverse stressors.

**Supplementary Information:**

The online version contains supplementary material available at 10.1007/s11356-022-23801-3.

## Introduction

Present-day marine plastic waste is often associated with aquaculture and fishing practises, or improper litter disposal (Galgani et al. 2015). Plastic is also considered a long-term source of toxic additives such as phthalates, which may leach from the plastic surface into the environment (Engler 2012). As a plastic softener compound, di-2-ethylhexyl phthalate (DEHP) has been added to polyvinyl chloride (PVC) for years (Erythropel et al. 2014). Despite the restricted use in the European Union (European Union Commission Regulation 2018/2005 2018), DEHP still represents almost 40% of the global plasticiser market (ECPI 2020). As a result, average concentrations between 0.145 (Sánchez-Avila et al. 2012) and 71.7 µg/L (Jebara et al. 2021) were detected in marine environments. The effects of DEHP on *Mytilus* spp. range from alterations in antioxidant and peroxisomal enzyme activities at high levels of 100–500 µg/L (Cancio et al. 1998; Orbea et al. 2002) to hormetic effects on the expression of oestrogen receptor-like (Mincarelli et al. 2021) and stress-related genes (Xu et al. 2021) when environmentally relevant concentrations are dosed. In fact, the nonmonotonic dose–response action of some endocrine active chemicals such as DEHP can provoke a stronger effect at low concentrations and inhibition at higher levels (Conolly and Lutz 2004; Do et al. 2012).

This scenario of increasing plastic pollution of aquatic environments coincides with an increase in atmospheric CO_2_ levels, which has already led to a reduction of oceanic surface water pH with respect to pre-industrial levels (IPCC 2021). Surface pH is predicted to decrease under all projected scenarios for the end of the century, and to a larger extent in high-latitude oceans, especially the Arctic Sea (Kwiatkowski et al. 2020). This may exacerbate naturally occurring pH fluctuations in coastal and near-shore habitats, to which intertidal species are adapted (Baumann and Smith 2018; Wolfe et al. 2020). Besides possible commercial repercussions of decreased oceanic pH on economically important calcifying organisms such as mussels (Mangi et al. 2018), short-to-medium term pH drop can also have a range of other consequences. In bivalves, a decrease in pH can alter immune responses (Bibby et al. 2008), affect calcification and energy metabolism-related gene expression (Hüning et al. 2013) and impact growth performances in larvae (Gazeau et al. 2010) and adults during gonadal ripening (Zhao et al. 2019).

Pollutants also frequently alter their toxicity depending on climate conditions, and chemicals often impact the ability of organisms to adapt to environmental fluctuations (Landis et al. 2014; Nikinmaa 2013). Low pH is reported to affect mussel biological responses when exposed in combination with pharmaceutical products (Mezzelani et al. 2021), heavy metals (Han et al. 2014) or illicit drugs (da Silva Souza et al. 2021). Likewise, the responses to plasticisers and other contaminants on mussels could be altered by concomitant changes in ocean chemistry with respect to CO_2_-induced ocean acidification, even considering pre-adaptation to pH-fluctuating current environments. Thus, it becomes extremely important to investigate the repercussions of these chemicals in altered pH conditions.

The scenario is further complicated by additional factors such as sex and reproductive status. These may affect both contaminant uptake and elimination, as well as biomarker levels and activities (Blanco-Rayón et al. 2020; Matozzo and Marin 2010). Moreover, natural differences in basal antioxidant levels between males and females could favour one sex over the other when coping with stressful environments (Gismondi et al. 2012; Sroda and Cossu-Leguille 2011). Thus, sex and reproductive status identification can be advantageous in analysing exposure experiment results.

For years, *Mytilus* spp. have been commonly used in biomonitoring programs worldwide or in ecotoxicological experiments (Laouati et al. 2021; Marigómez et al. 2013), as these molluscs are considered key biomonitors for their habitats (Markert et al. 2003). Mussels are also used as distinctive indicators of health and food safety because of their position in the food chain and their close relationship with the human diet (Chiesa et al. 2018; Van Cauwenberghe and Janssen 2014). Here, we conduct a multi-factor investigation into the blue mussel, *M. edulis*, gene expression under a pH 7.7 scenario combined with DEHP additive exposure in environmentally relevant concentrations, while considering sex-based and reproductive differences. Genes for superoxide dismutase (*sod*) and catalase (*cat*) were chosen as part of the antioxidant enzyme system due to their coordinated roles in reducing superoxide anion O_2_^−^, a reactive oxygen species (ROS, Regoli and Giuliani 2014). Heat shock protein 70 (*hsp70*) was selected as a biomarker of stress responsive to environmental perturbation (Encomio and Chu 2005; Lewis et al. 1999) and xenobiotic exposure (Franzellitti and Fabbri 2005; Koagouw et al. 2021). Genes coding for carbonic anhydrase 2 (*CA2*), oestrogen-related receptor (ERR, *MeER1*) and oestrogen receptor (ER, *MeER2*) were chosen as they are associated with biomineralisation, pH homeostasis and reproductive cycle, whose expression can be affected by oestrogenic compounds (Balbi et al. 2016; Ciocan et al. 2010; Nagasawa et al. 2015).

## Materials and methods

### Experimental design

Adult blue mussels (*n* = 180; length mean ± standard deviation = 4.9 cm ± 0.5 cm) were collected from the suspended ropes farm of Cromarty Mussels, Ltd. in Cromarty Firth, Scotland, UK (57.40.741 N 4.06.062 W) in January 2020 and transported to the aquarium facilities of the University of Hull. Thirty mussels for each of the 6 treatments were randomly divided into 6 4-L continuously aerated glass tanks, for a total number of 5 mussels for each replicate tank at a density of 1 mussel per 0.8 L (Supplementary Fig. 1). They were kept for acclimation for 12 days in artificial saltwater (Premium REEF-Salt, Tropical Marine Centre, Chorleywood, UK) in a climate-controlled room at photoperiod 10:14 light:dark, salinity of 35 psu, pH of 8.1 units and temperature of 9 °C, in line with the natural environmental conditions in Cromarty Firth at the time of collection. The number of 30 individuals was chosen for each exposure treatment to ensure an adequate number of animals for each sex. After the acclimation period, mussels were exposed for 7 days to two different pH levels (8.1 and 7.7) and three concentrations of DEHP (0, 0.5 and 50 µg/L), for a final yield of six experimental treatments (CTRL, LOW pH, LOW DEHP, LOW DEHP LOW pH, HIGH DEHP, HIGH DEHP LOW pH). For the pH exposure, a total decrease of 0.4 units for the 7.7 low pH treatment was chosen considering the projected range for ocean acidification conditions for the year 2100 (IPCC 2021). DEHP exposures of 0.5 and 50 µg/L were chosen from the literature, considering the levels found in marine coastal waters (Jebara et al. 2021; Sánchez-Avila et al. 2012). The 7-day DEHP exposure was chosen accounting for the non-persistency of the plasticiser in the environment (Staples et al. 1997), with a half-life of approximately 0.35–3.5 days for surface water and sediments in aerobic conditions (Peterson and Staples 2003). Mussels were not fed during the exposure and artificial saltwater was prepared the day before each water change, to allow the water temperature to adjust to the controlled room. Water was changed every second day and DEHP was dosed right after (i.e. days 1, 3 and 5) from a stock solution of 1 mg/mL DEHP (≥ 99.5% purity, Sigma Aldrich®, Gillingham, UK) in ethanol. The 7.7 pH values were adjusted by mixing seawater with small amounts of CO_2_-saturated water over the course of the exposure week (Nardi et al. 2017; Schulz et al. 2013). Temperature, pH and salinity were measured daily (Supplementary Table 1) with a digital thermometer (Amarell Thermometer, Kreuzwertheim, Germany), a pH meter (Jenway, Bibby Scientific Limited, Stone, UK) and a digital seawater refractometer (Hanna Instruments, Woonsocket, USA). Alkalinity was measured twice a week with a HI 84531 mini titrator (Hanna Instruments, Woonsocket, USA). After 7 days of exposure, tissues from gonads were collected for molecular and histological analyses (unlicensed animal ethics approval; reference no #U080/FEC_2021_11, University of Hull). Approximately 1.0 cm^2^ of left gonad tissue was immersed in 1 mL neutral-buffered 10% formalin solution (Sigma Aldrich, Gillingham, UK) at room temperature for histological observations. The same gonadal amount was dissected and preserved in 1 mL RNAlater® stabilisation solution for gene expression analysis (Thermo Fisher Scientific, Loughborough, UK) and stored at − 80 °C.

## Histological analysis

**Fig. 1:**
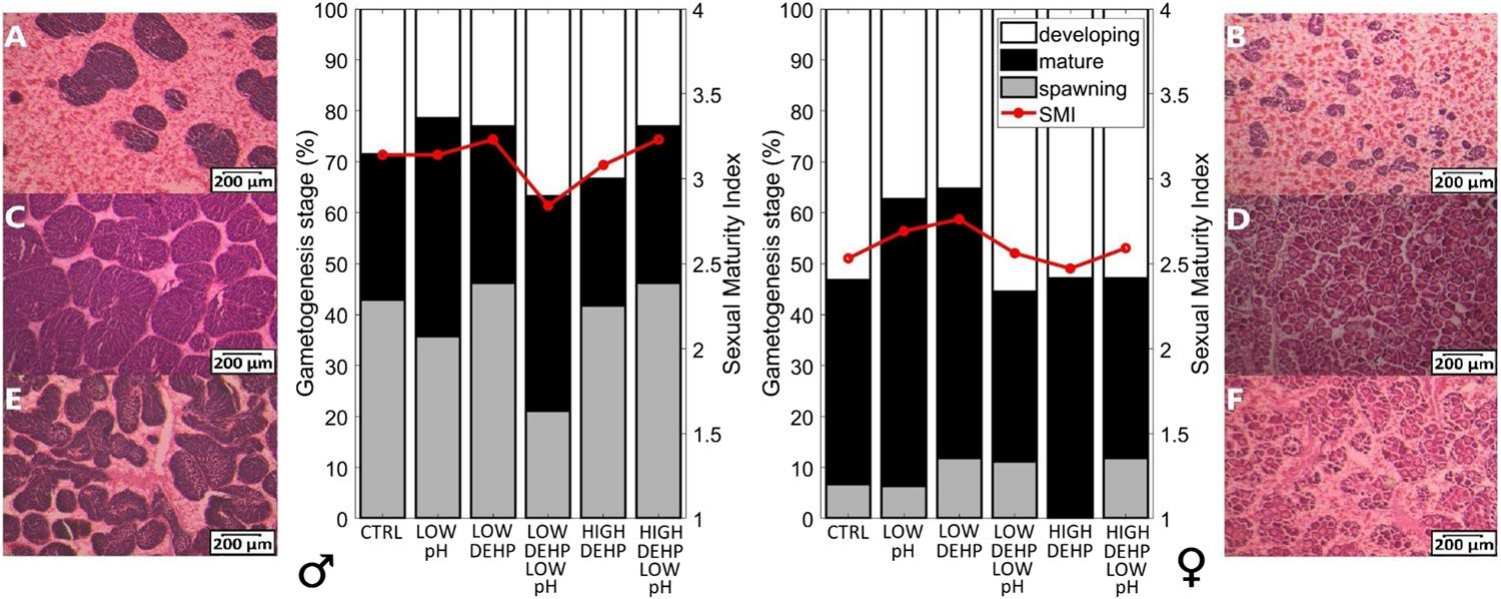
Percentage of each gametogenesis stage and sexual maturity index (SMI) of males (left) and females (right) in CTRL (*n* = 14 (males), 15 (females)), LOW pH (*n* = 14 (males), 16 (females)), LOW DEHP *(n* = 13 (males), 17 (females)), LOW DEHP LOW pH (*n* = 19 (males), 9 (females)), HIGH DEHP (*n* = 18 (males), 11 (females)) and HIGH DEHP LOW pH (*n* = 17 (males), 13 (females)). Gametogenesis stages of 10-µm gonadal tissue sections stained with haematoxylin and eosin are displayed alongside. Developing status in males (**A**) and females (**B**), mature gonads in males (**C**) and females (**D**), spawning stage in males (**E**) and females (**F**). Scale bars represent 200 µm. Images were modified for brightness and contrast

Gonad samples were washed with phosphate-buffered saline (PBS) and dehydrated with increasing ethanol concentrations (70, 90, 100%). Samples were then cleared with Histoclear II® (National Diagnostics, Atlanta, USA) and embedded in paraffin wax. Tissue sections of 10-µm gonads were cut on an automatic microtome (Thermo Fisher Scientific, Loughborough, UK) and stained using haematoxylin and eosin solutions (Sigma Aldrich, Schnelldorf, Germany). To conduct blind observations, sample slides were coded before the microscope identification. Sex and reproductive status were assessed under a light microscope following Seed (1969): (i) development (Fig. 1A and B); (ii) mature stage (Fig. 1C and D); (iii) spawning (Fig. 1E and F). Each stage was categorised by a maturity factor (MF): (i) MF = 1 for resting or spent gonad; (ii) MF = 2, developing gonads; (iii) MF = 3, mature gonads; (iv) MF = 4, spawning gonads. Then, the sexual maturity index (SMI) was calculated according to the equation established by Siah et al. (2003): *SMI* = *Σ (proportion of each stage* × *maturity factor)*.

## Gene expression analysis

For the gene expression analysis, 8 female and 8 male gonads (approx. 10 mg of gonad tissue, *n* = 96) were selected randomly and blindly coded, and total RNA was extracted from gonadal tissues using the High Pure RNA Tissue Kit (Roche Applied Science, Burgess Hill, UK), including a 15-min DNase I treatment at 25 °C. Then, cDNA templates were synthesised using 200 units of Invitrogen™ SuperScript™ II Reverse Transcriptase (Fisher Scientific, Loughborough, UK). Since *M. edulis* frequently hybridises with *M. galloprovincialis* throughout its distribution area (Simon et al. 2019), species and potential hybrids were identified by PCR and agarose gel electrophoresis for the non-repetitive region of the *Mytilus foot protein 1* (*mfp-1*). Primer set *Me15* and *Me16* were taken from Inoue et al. (1995), and used in combination with 12.5 µL of PCRBIO Taq Mix Red (containing 6 mM MgCl_2_, 2 mM dNTPs, PCR BioSystems, London, UK), 1.25 µL of cDNA and the following thermal conditions: pre-heating to 95 °C for 5 min, followed by 40 cycles of 1 min at 95 °C, 1 min at 60.5 °C and 1 min at 72 °C and a final extension step of 10 min at 72 °C.

Primer sequences for qPCR gene expression analysis were taken from the literature: *elongation factor-1 alpha (EF1α)* (GenBank accession no. **AF063420**), *18SrRNA (Me18S)* (**L33448**) and *28SrRNA (Me28S)* (**Z29550**) from Ciocan et al. (2011); s*uperoxide dismutase (sod)* (**AJ581746**), *heat shock protein 70 (hsp70)* (**AF172607**) and *oestrogen receptor 1 (MeER1*) (**AB257132**) from Mincarelli et al. (2021); *carbonic anhydrase 2 (CA2)* (**LK934681.1**) from Balbi et al. (2016); and *oestrogen receptor 2 (MeER2)* (**AB257133**) from Puinean et al. (2006). Additionally, new primers were designed using Primer3 (http://primer3.ut.ee/) from the published sequence for *catalase (cat)* (**AY580271**)*.* Only primer efficiencies between 90 and 110% were accepted, in accordance with the MIQE guidelines (Bustin et al. 2009). Primer details are provided in Supplementary Table 2. *Me18S*, *Me28S* and *EF1α* were chosen as they represent suitable reference genes during mussel gametogenesis and exogenous oestrogenic exposures (Cubero-Leon et al. 2012). *EF1α* and *Me28S* genes were furthermore chosen for normalisation of the final dataset using the 2^−ΔCt^ and 2^−ΔΔCt^ methods (Schmittgen and Livak, 2008), being considered the most stable combination by RefFinder software and Kruskal–Wallis test (*Me18S* KW-H = 2.1865, *p* = 0.8228; *Me28S* KW-H = 3.0456, *p* = 0.6929; *EF1α* KW-H = 7.697, *p* = 0.1737). qPCR reactions were performed on a CFX96 Real-Time PCR Detection System (Bio-Rad, Hemel Hempstead, UK) using 10 µL of qPCRBIO SyGreen Mix Lo-ROX (PCRBioSystem, London, UK), 7.5 µL molecular-grade water, 1 µL of each primer and 0.5 µL cDNA. Final primer concentrations are given in Supplementary Table 2. Thermal cycling was as follows: 95 °C for 2 min, 40 cycles of 95 °C for 5 s, 60 °C for 30 s and 72° C for 1 min. Template negatives were included alongside samples.

## Statistical analysis

The histology dataset was analysed with general ordered logit with partial proportional odds model, to predict the dependent variable “gametogenesis stage”, assuming “DEHP”, “pH” and “sex” as independent variables, after verifying the rejection of the proportional odds assumption (test of Parallel Lines, *ordinal* package, Christensen 2019). Model uncertainty was assessed by comparing ΔAICc values and Akaike weights. Model selection was carried out in RStudio with the *AICcmodavg* package (Mazerolle 2020) in R 4.0.3 (CRAN). The model was estimated using the *vglm* function (VGAM package, Yee 2010), calculating the *p* error probability by comparing the *z*-value against the standard normal distribution.

Permutation multivariate analysis of variance (PERMANOVA, Anderson 2014) was used in RStudio (*vegan* package, Oksanen et al. 2013), to test the effects of pH, DEHP, sex, gametogenesis stage on the 2^−ΔΔCt^ values of the stress-related (*sod*, *cat* and *hsp70*), oestrogen receptor-like (*MeER1* and *MeER2*) and pH homeostasis (*CA2*) gene expression using Bray–Curtis distance and 9999 permutations (Anderson 2014). Factors “sex” (males or females) and “stage” (developing, mature, spawning) were added for the PERMANOVA analysis to underline sex- and gametogenesis-driven differences between the treatments. Possible outliers were identified by Grubb’s test (Grubbs 1969) and outlier values beyond the significance level of *α* = 0.05 were rejected (Burns et al. 2005). Pairwise multilevel comparison with Benjamini and Hochberg *p*-adjustment was used to compare male and female groups. Moreover, regarding the effect of different treatments on the expressions of each gene, an additional non-parametric Scheirer-Ray-Hare (SRH, *rcompanion* package, Mangiafico 2017) test was used on the 2^−ΔCt^ values, after verifying non-normal distribution (Shapiro–Wilk test) and homogeneity of variances (Levene’s test). Statistical significance was set to *p* < 0.05. All graphs were created using MATLAB R2021a.

## Results

### Histology results to determine gametogenesis stages

The dataset analysed in this experiment consisted of 46% females and 54% males, an approximately regular 1:1 proportion for sex ratio in a mussel population. The most parsimonious model (using only “sex” as predictor variable, Supplementary Table 3) showed a predictably significant difference between males and females in the transition from developing to the more advanced stages (mature and spawning, *p* sex = 0.01, *z*-value = 2.49), with male SMI being overall higher than that of females (Fig. 1). The effect of the predictors “pH” and “DEHP” was also tested, confirming no significant effect for either of them (*p* > 0.05).

### Molecular analysis

Molecular species identification confirmed that the sampled population consisted of *M. edulis* and neither hybrids with *M. galloprovincialis* nor other *Mytilus* species were present (Supplementary Fig. 2).

**Fig. 2:**
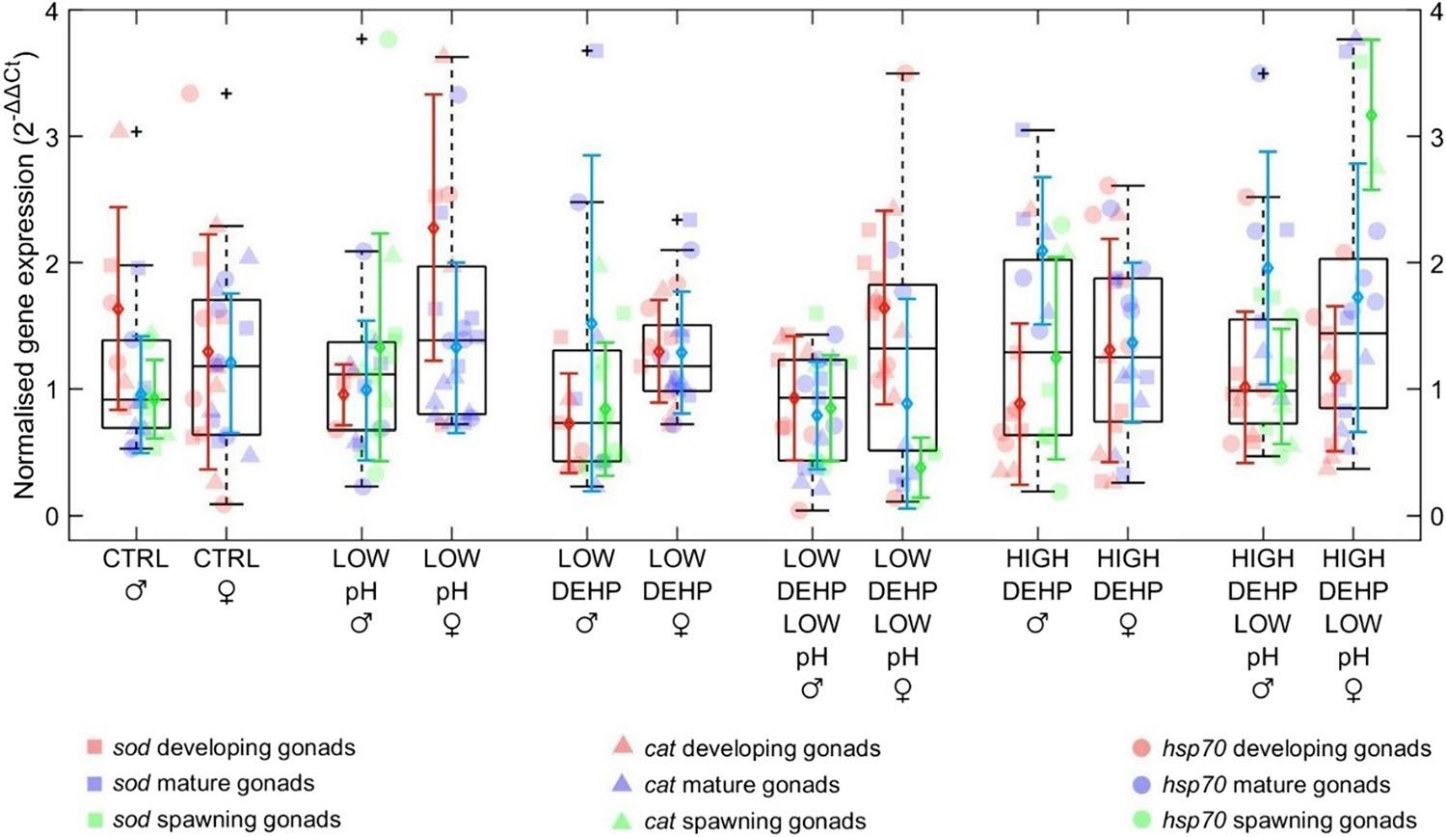
Boxplots showing stress-related (*sod*, *cat*, *hsp70*) gene expression in males and females, *n* = 6–8, considering sex and gametogenesis stage (developing. mature, spawning) of the gonads. Excluded outliers are not shown, while the furthest accepted values are identified by black plus symbols. Datapoints, means and standard deviations for each gametogenesis stage are displayed in red (developing gonads), blue (mature gonads) and green (spawning gonads). Different genes are displayed in squares (*sod*), triangles (*cat*) and circles (*hsp70*). Abbreviations are control (CTRL), low pH (LOW pH), low DEHP concentration (LOW DEHP), low DEHP at low pH (LOW DEHP LOW pH), high DEHP concentration (HIGH DEHP) and high DEHP at low pH (HIGH DEHP LOW pH). Significant factors in PERMANOVA are sex *p* < 0.01 (**), DEHP*stage *p* < 0.01 (**), pH*DEHP*stage *p* = 0.09 and DEHP*sex*stage *p* = 0.09

Using “pH”, “DEHP”, “sex” and “gametogenesis stage” as predictors for stress-related gene expression in PERMANOVA analysis, we observed both significant differences between sexes (*p* sex = 0.001, *F* = 6.97, Fig. 2 and Table 1) and an effect of DEHP exposure in interaction with gametogenesis stage (*p* DEHP*stage = 0.005, *F* = 2.79, Fig. 2). A slight but non-significant trend was observed for the combination of pH, DEHP and stage (*p* pH*DEHP*stage = 0.09, *F* = 1.76, Fig. 2) and DEHP, sex and stage (*p* DEHP*sex*stage = 0.09, *F* = 1.82, Fig. 2). Overall, stress-related gene expression was higher in females than in males and higher in male mature gonads than in developing ones exposed to the high DEHP treatments. In females, an opposite trend was observed in the groups co-exposed to the two stressors, with a downregulation in the LOW DEHP LOW pH and an upregulation in the HIGH DEHP LOW pH following the progression of gonadal maturation.

**Table 1:** Pairwise multilevel comparisons of the stress response (*sod*, *cat*, *hsp70*) between males and females in the same treatments. Significant differences and related *p* values are shown in italics

Treatment males	Treatment females	F.model	*p*
CTRL	CTRL	0.36	0.77
LOW pH	LOW pH	2.09	0.12
*LOW DEHP*	*LOW DEHP*	*3.46*	*0.05*
LOW DEHP LOW pH	LOW DEHP LOW pH	1.08	0.37
HIGH DEHP	HIGH DEHP	2.62	0.09
HIGH DEHP LOW pH	HIGH DEHP LOW pH	1.29	0.29

**Fig. 3:**
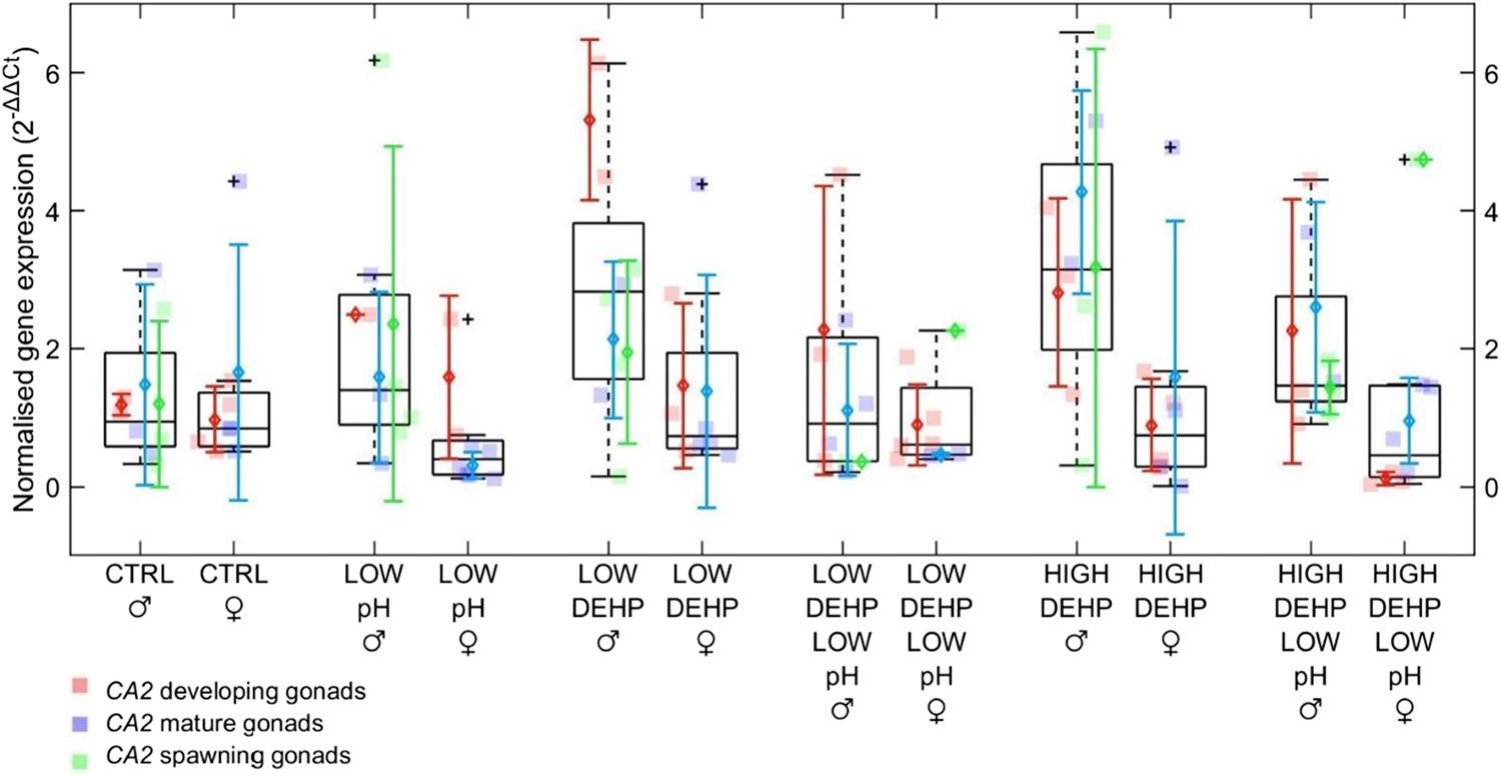
Boxplots showing *CA2* gene expression in males and females, *n* = 8, considering sex and gametogenesis stage (developing, mature, spawning) of the gonads. The furthest accepted values are identified by black plus symbols. Datapoints, means and standard deviations for each gametogenesis stage are displayed in red (developing gonads), blue (mature gonads) and green (spawning gonads). Abbreviations are control (CTRL), low pH (LOW pH), low DEHP concentration (LOW DEHP), low DEHP at low pH (LOW DEHP LOW pH), high DEHP concentration (HIGH DEHP) and high DEHP at low pH (HIGH DEHP LOW pH). PERMANOVA error probabilities are sex *p* < 0.001 (***), sex*stage *p* = 0.02 (**) and pH *p* = 0.06

Expression of the pH responsive *CA2* gene was only slightly modulated by low pH (PERMANOVA *p* pH = 0.06, *F* = 2.80, Fig. 3), with a trend towards downregulation of the gene in females in the pH 7.7 treated groups (Supplementary Fig. 12). Better predictors for variance in *CA2* expression were sex (*p* sex < 0.001, *F* = 13.8, Fig. 3 and Table 2) and the interaction term of sex and gametogenesis status (*p* sex*stage = 0.02, *F* = 3.72, Fig. 3). As expected, DEHP treatments did not have any effect on the expression of this acid–base regulatory enzyme.

**Table 2:** Pairwise multilevel comparisons of the *CA2* response between males and females in the same treatments. Significant differences and related *p* values are shown in italics

Treatment males	Treatment females	F.model	*p*
CTRL	CTRL	0.01	0.98
*LOW pH*	*LOW pH*	*6.29*	*0.02*
LOW DEHP	LOW DEHP	1.90	0.18
LOW DEHP LOW pH	LOW DEHP LOW pH	0.30	0.65
*HIGH DEHP*	*HIGH DEHP*	*3.21*	*0.04*
*HIGH DEHP LOW pH*	*HIGH DEHP LOW pH*	*5.13*	*0.04*

**Fig. 4:**
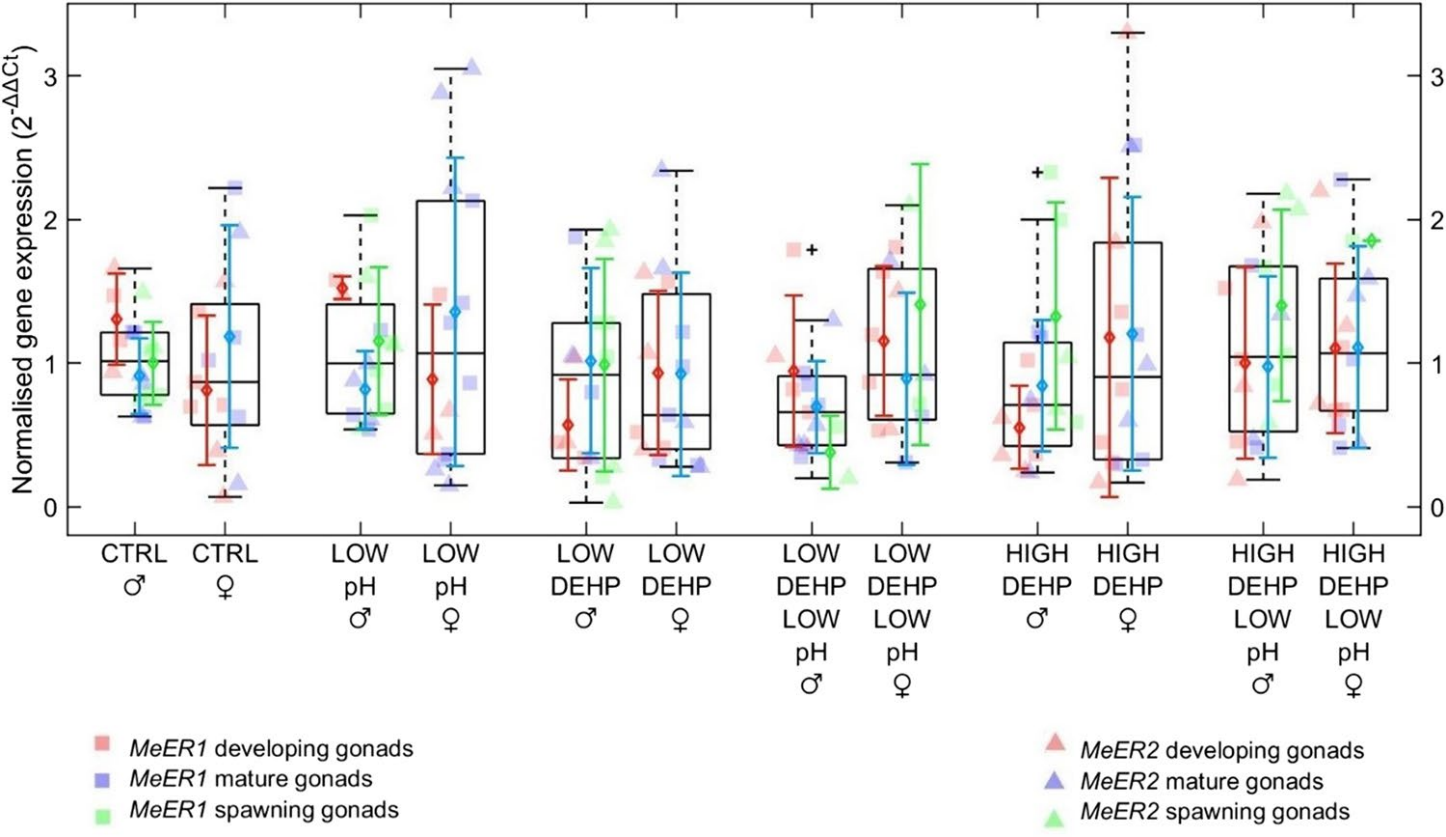
Boxplots showing oestrogen receptor-like (*MeER1*, *MeER2*) gene expression in males and females, *n* = 5 to 8, considering sex and the gametogenesis stage (developing. mature, spawning) of the gonads. Excluded outliers are not shown, while the furthest accepted values are identified by black plus symbols. Datapoints, means and standard deviations for each gametogenesis stage are displayed in red (developing gonads), blue (mature gonads) and green (spawning gonads). Different genes are displayed in squares (*MeER1*) and triangles (*MeER2*). Abbreviations are control (CTRL), low pH (LOW pH), low DEHP concentration (LOW DEHP), low DEHP at low pH (LOW DEHP LOW pH), high DEHP concentration (HIGH DEHP) and high DEHP at low pH (HIGH DEHP LOW pH). No significant PERMANOVA differences were found

Finally, neither the drop to pH 7.7 nor the DEHP exposure did have any consequences on *MeER1* and *MeER2* expression, and neither did sex nor the gametogenesis status apart from a significant difference between sexes in the LOW DEHP LOW pH group (Fig. 4, Table 3).

**Table 3:** Pairwise multilevel comparisons of the oestrogen receptor-like response (*MeER1*, *MeER2*) between males and females in the same treatments. Significant differences and related *p* values are shown in italics

Treatment males	Treatment females	F.model	*p*
CTRL	CTRL	0.80	0.41
LOW pH	LOW pH	0.48	0.55
LOW DEHP	LOW DEHP	0.41	0.66
*LOW DEHP LOW pH*	*LOW DEHP LOW pH*	*3.03*	*0.05*
HIGH DEHP	HIGH DEHP	1.88	0.16
HIGH DEHP LOW pH	HIGH DEHP LOW pH	0.27	0.71

Considering the single gene expression (2^−ΔCt^ values) in either females or males, no particular effect was found (Supplementary Fig. 3–14). Details of mRNA expression levels of each gene are provided in Supplementary Tables 4 and 5.

## Discussion

### Histology results to determine gametogenesis stages

Histology observations of the gonadal gametogenesis status revealed male and female *M. edulis* to be in advanced stages of the gametogenesis cycle, with the majority of females late developing or mature and males ripening or spawning. Overall, there was a difference of *ca.* one point between their sexual maturity indices. Such an asynchrony between sexes in ripeness proportions was already shown in *M. barbatus* (Mladineo et al. 2007), as well as different timing of spawning events for *M. galloprovincialis*, which nonetheless should not preclude successful fertilisation (Azpeitia et al. 2017). This difference could also be related to the scheme of scientific classification of the different stages of the gonadal cycle, for which female sex often appears to be slightly behind males. However, it is also possible that the production of spermatozoa is faster than the ova, due to the large yolk reserves of the latter (Seed 1969).

By itself, DEHP was observed not to induce any severe modifications of the gametogenesis cycle, as we have previously found in blue mussels from Filey, North Yorkshire (Mincarelli et al. 2021). Similarly, no alterations of the gametogenesis cycle seemed to be present after 7 days of exposure to low pH in either sex. This could suggest that the stimulation of gonadal maturation is not responsive to acidic pH but could possibly be more sensitive to alkaline ones, as shown for sea snails’ oocytes (Aquino De Souza et al. 2009). On the other hand, mussels inhabit naturally pH-fluctuating environments, often influenced by variable immersive time and freshwater input. This could result in a heightened tolerance and adaptation to local pH fluctuations, possibly resulting in no immediate repercussions for the reproductive cycle. However, according to Zhao et al. (2019), exposure to pH 7.7 for 40 days decreased the percentage of *M. senhousia* spawning gonads by shifting the energy budget towards more essential physiological processes such as acid–base regulation. This suggests that a higher susceptibility to prolonged acidified conditions in the final gametogenesis stages is possible.

## Expression analysis of stress-related genes

Regarding the stress response at the molecular level, represented in this experiment by the expression of the genes *sod*, *cat* and *hsp70*, the PERMANOVA analysis showed a significant influence of sex and of DEHP exposure in interaction with the gametogenesis stage. In males, developing gonads showed lower levels with respect to mature ones in the groups exposed to the high concentration of DEHP. For females, a higher stress-related gene expression was observed overall compared to males. Similar results were noted in crustaceans *Pachygrapsus marmoratus* and *Daphnia magna*, which were observed to have higher levels of stress-related heat shock proteins in female individuals (Madeira et al. 2012; Mikulski et al. 2011), suggesting a different adaptive control of the HSP system in females, which could possibly allow them to be more resilient to stressed conditions than males (Gismondi et al. 2012). Furthermore, DEHP exposure resulted in significantly altering the stress response depending on gametogenesis stages. Levels and activities of oxidative stress biomarkers are observed to vary during the annual reproductive cycle of bivalves (Jarque et al. 2014; Wilhelm Filho et al. 2001). Therefore, the gametogenesis state could have contributed to the basal antioxidant levels and their reaction to contaminants, as reported in González-Fernández et al. (2016), where activities of CAT and glutathione peroxidase (GPx) were noticed to be affected by the chemical fluoranthene only during the gonadal resting period. This may also be related to the energy allocation that varies during reproductive and resting periods and could be affected by energy-demanding stress responses (Madeira et al. 2012). Additionally, Yu et al. (2021) observed that the expression of HSP90 isoforms was significantly higher in gonads of the scallop *Chlamys farreri* compared to non-reproductive tissues, suggesting an involvement of these proteins the gametogenesis process.

The mild changes in expression in response to pH 7.7 exposure seem to be in line with the hypothesis that organisms from habitats characterised by fluctuating conditions such as coastal and near-shore environments could be less sensitive and more tolerant to variations in water pH. In support of this theory, early stages of *Strongylocentrotus purpuratus* purple sea urchins from naturally low and variable pH habitats showed adaptive calcification strategies and absence of a generalised stress response when exposed to high *p*CO_2_ (Evans et al. 2013). Nonetheless, other alterations from ocean acidification in the medium-long term on the immune system (Beesley et al. 2008), feeding ability (Xu et al. 2020), growth (Michaelidis et al. 2005) and other physiological processes (Navarro et al. 2013) cannot be ruled out, as some mussel individual traits and population characteristics seem to be influenced by habitat parameters such as intertidal height and shore orientation (Barbosa et al. 2021). Juvenile stages of mussels are reported to be able to cope with decreased surface water pH if food supply is sufficient (Thomsen et al. 2013); thus, an effect of a low pH exposure in the long term in combination with nutrient scarcity is plausible, considering that the reaction to stress is a dynamic and integrated response involving molecular, cellular and physiological processes within the organisms (Sokolova et al. 2011). In fact, Guo et al. (2021) doubted the survival of *M. edulis* under multiple stressors combined with elevated *p*CO_2_. Individuals were observed to moderately adapt and tolerate ocean acidification by increasing the synthesis of ATP and reallocating the energy to gills and haemocytes. Nonetheless, the excessive energy consumption was not compensated in hypercapnic environments, eventually leading to increased mortality.

## Expression analysis of pH homeostasis gene

In this study, we analysed the expression of *CA2*, a gene part of the family of carbonic anhydrases (CAs), which control the intra- and extracellular pH homeostasis catalysing the reversible carbonic hydration from CO_2_ to bicarbonate (Richier et al. 2011). Here, we found a significant difference in *CA2* expression based on sex, considered alone and in combination with the gametogenesis status. Higher levels of *CA2* 2^−ΔΔCt^ values were found in male groups with respect to females, and possibly associated with metabolic profiles, hormonal state or fitness strategies (Ji et al. 2013; Mikulski et al. 2011; Wong et al. 2014). It was reported that CA activity was found at different levels during the life cycle of *M. edulis*, being highest at the end of the developmental stages (Medaković 2000). Interestingly, in Wang et al. (2017), expression of the *CAII-1* gene in *Crassostrea gigas* exposed to low pH was downregulated only in male gonads, in contrast with a significant upregulation in other non-reproductive tissue samples. Together, these findings suggest a tissue-specific regulation of this metalloenzyme and a potential link to the reproductive system status which could explain the differences we observed between sexes at different stages of the gametogenesis cycle.

With the exception of the LOW DEHP LOW pH treatment, males and females exposed to pH 7.7 (LOW pH and HIGH DEHP LOW pH) statistically differed in their *CA2* expression. We also found that the expression of *CA2* was slightly modulated by low pH, with a general downregulation of the enzyme especially in females. This contrasted with Wäge et al. (2016), where a short exposure to low pH induced an upregulation of *CA* in polychaete worms *Platynereis dumerilii*. In agreement with our results, pH drop is known to induce downregulation of this enzyme and loss of shell structural integrity in several calcifying species including molluscs (Fitzer et al. 2014; Zebral et al. 2019). This might be caused by a compensatory strategy in response to the alteration of the acid–base balance in the body fluid from hypercapnic conditions. Downregulation of *CA* in clam *Panopea globosa* larvae was explained as a feedback response to its decreased activity at low pH (López-Landavery et al. 2021), probably caused by enzyme denaturation or lowered efficiency (Sun et al. 2016).

Chemical compounds such as metals in molluscs and crustaceans are known to alter CA activities (Lionetto et al. 2006; Skaggs and Henry 2002), but in our experiment, DEHP exposure did not. This could be possibly related to the metal-binding affinities of CAs and to the more effective osmo-, ionoregulatory and acid–base disruption ability of certain metals compared to other chemicals (Bianchini et al. 2005; Lionetto et al. 1998). However, Balbi et al. (2016) found an effect on carbonic anhydrases in *M. galloprovincialis* larvae exposed to 1–10 mg/L of the oestrogenic chemical bisphenol A, often used as an additive in polycarbonate plastic production. This could suggest a greater effect of endocrine disruptive chemicals at higher concentrations on the vulnerable first phases of development, when the biomineralisation process is still at early stages and thus more sensitive.

## Expression analysis of oestrogen receptor-like genes

Finally, we analysed the expression of *MeER1* and *MeER2*, two genes part of the oestrogen receptor-like system and biomarkers of reprotoxicity. As already noted with respect to the gonadal state histologically investigated here, a drop to pH 7.7 did not induce any consequences on their expressions. This reinforces the hypothesis that the reproduction cycle of mussels is less sensitive to acidic environments. Likewise, DEHP exposure did not influence the expression of *MeER1* and *MeER2*, in contrast to other studies that reported an effect of xenobiotics such as the plastic additive bisphenol A (Balbi et al. 2016) or the pharmaceutical metformin (Koagouw and Ciocan 2018) on the expression of oestrogen receptor-like genes. Interestingly, DEHP did not elicit a response on oestrogen receptor-like gene expression in either sex. This result contrasts with the preceding findings of our team in a previous experiment (Mincarelli et al. 2021) where mussels were co-exposed to DEHP and high temperature for 1 week. In that case study, DEHP exposure significantly affected the oestrogen receptor-like pathway, especially in developing females’ *MeER1*. Some factors that may account for these differences between the two experiments are that mussels originated from two different populations (North Yorkshire in contrast to Cromarty Firth), years (2018 and 2020), seasons (early in contrast to late winter) and the related gametogenesis gonadal status. Natural variation in the oestrogen receptor-like physiological conditions could be based on annual and seasonal contexts, as already noted for *M. edulis MeER2* expression (Ciocan et al. 2010). Similarly, variable expression of oestrogen receptor-like genes was found during *M. galloprovincialis* ovarian cycle (Agnese et al. 2019) and throughout larval development (Balbi et al. 2016). In this case, the initial hypothesis of this experiment that the action of certain stressors could be affected by the reproductive cycle did not find confirmation, as we found no significant influence by the gametogenesis stage on the gene expression of oestrogen receptor-like response.

## Conclusion

In conclusion, we found that the response of *M. edulis* to different stressors (pH 7.7 conditions and two environmentally relevant concentrations of the plasticiser DEHP for 1 week) is strongly dependent on sex and developmental status of gonads. As shown before, sex differences were observed for genes involved in the stress response and acid–base balance, underlying the possibility of a better adaption of either sex in future climate conditions. This paper lends support to the need of identifying sex and gonadal maturation stages in mussels when measuring multiple stressor responses, which is necessary in a scenario of plastic-polluted and acidified oceans.

## Supplementary Information

Below is the link to the electronic supplementary material.

Supplementary file1 (DOCX 1.14 MB)

## Data Availability

Details of mRNA expression levels of each gene are provided in the Supplementary Material. Other original data and materials from this paper could be available upon reasonable request.
