# Supplementary material for: Sex and gametogenesis stage are strong drivers of gene expression in Mytilus edulis exposed to environmentally relevant plasticiser levels and pH 7.7
Source: Environ Sci Pollut Res Int. 2022 Nov 2;30(9):23437–49. doi: 10.1007/s11356-022-23801-3 (PMC9938808; doi:10.1007/s11356-022-23801-3)
Supplement: Supplementary file 1 — Supplementary file1 (DOCX 1.14 MB) [file 11356_2022_23801_MOESM1_ESM.docx]

## Supplementary Material

**to the manuscript**

**Sex and gametogenesis stage are strong drivers of gene expression in *Mytilus edulis* exposed to environmentally relevant plasticiser levels and pH 7.7**

Luana Fiorella Mincarelli^1^, Emma C. Chapman^1^, Jeanette M. Rotchell^1^, Alexander P. Turner^2^, Katharina C. Wollenberg Valero^1*^

^1^ Department of Biological and Marine Sciences, University of Hull, Hull HU6 7RX, United Kingdom

^2^Department of Computer Science, University of Nottingham, NG8 1BB, United Kingdom

*corresponding author: k.wollenberg-valero@hull.ac.uk

**Contains:**

14 Supplementary Figures

5 Supplementary Tables

**
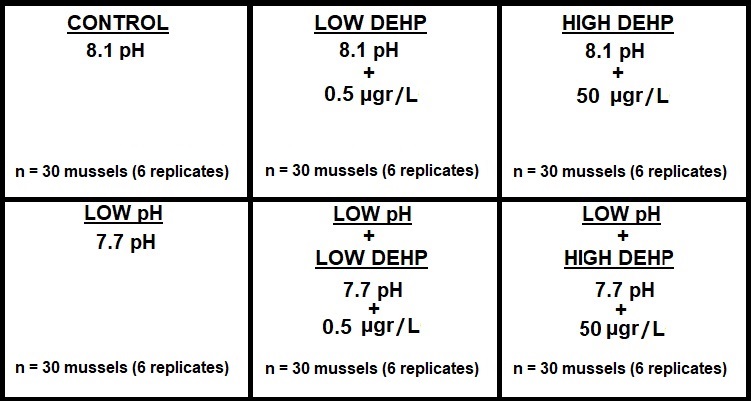
**

**Supplementary Fig. 1** Experimental design for the 7-day exposure with the chosen parameters for pH (8.1 and 7.7 units) and DEHP (0, 0.5 and 50 μg/L)


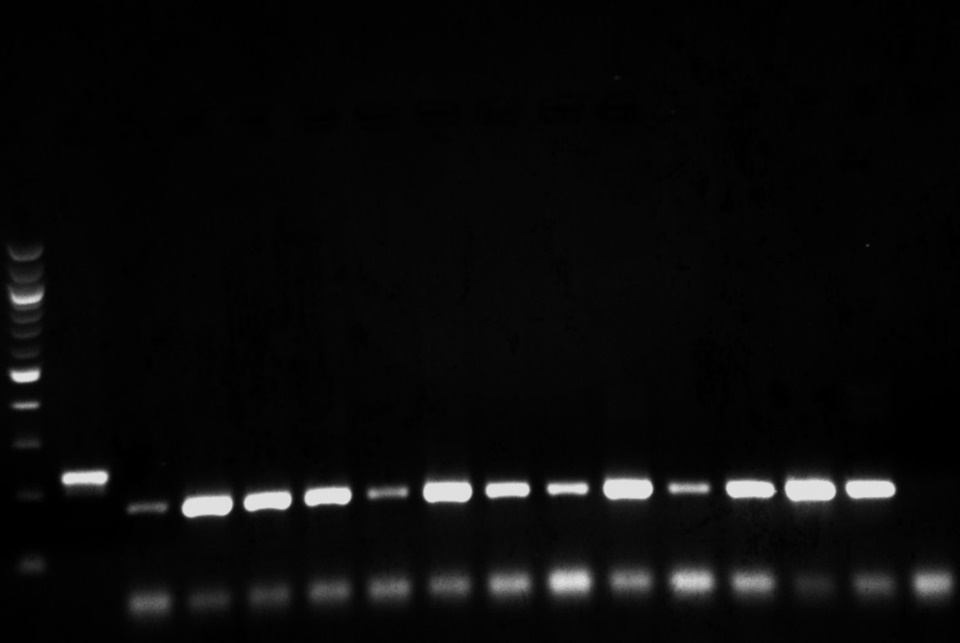


# Supplementary Fig. 2 Agarose gel (2%, GelRed™, 80V) showing from left to right: NeB DNA ladder, 100 bp; 235 bp positive control; 180 bp fingerprints of the *mfp-1* region (*M. edulis*); negative control (water)

##
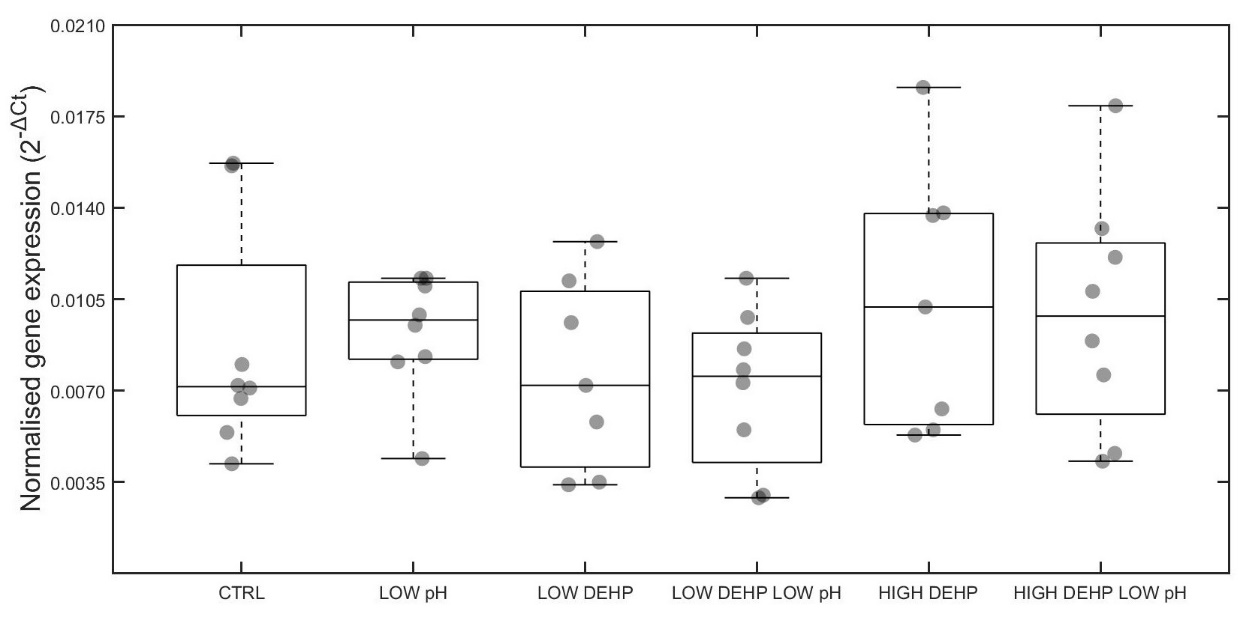


## Supplementary Fig. 3 Boxplot graphs showing *sod* mRNA expression in males, n= 7-8. Abbreviations are control (CTRL), low pH (LOW pH), low DEHP concentration (LOW DEHP), low DEHP at low pH (LOW DEHP LOW pH), high DEHP concentration (HIGH DEHP) and high DEHP at low pH (HIGH DEHP LOW pH). Each datapoint in grey represents the normalised gene expression as 2^-ΔCt^ values. Values are also reported in detail in Supplementary Table 4

##
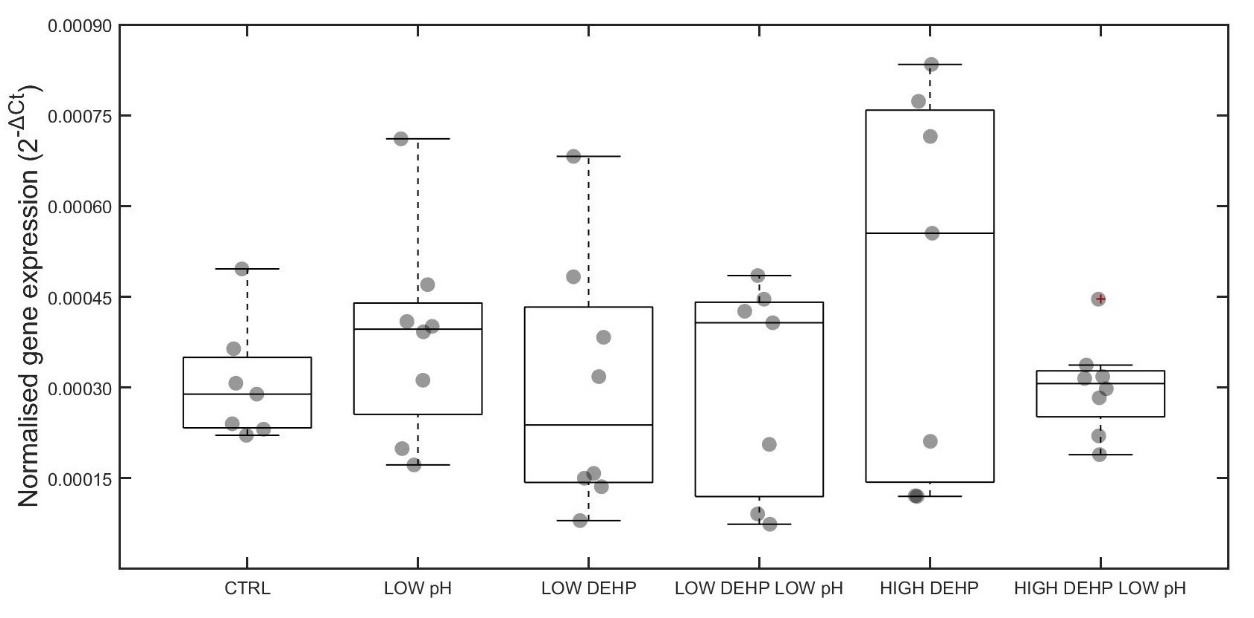


# Supplementary Fig. 4 Boxplot graphs showing *cat* mRNA expression in males, n= 7-8. Abbreviations are control (CTRL), low pH (LOW pH), low DEHP concentration (LOW DEHP), low DEHP at low pH (LOW DEHP LOW pH), high DEHP concentration (HIGH DEHP) and high DEHP at low pH (HIGH DEHP LOW pH). Each datapoint in grey represents the normalised gene expression as 2^-ΔCt^ values. Values are also reported in detail in Supplementary Table 4

#
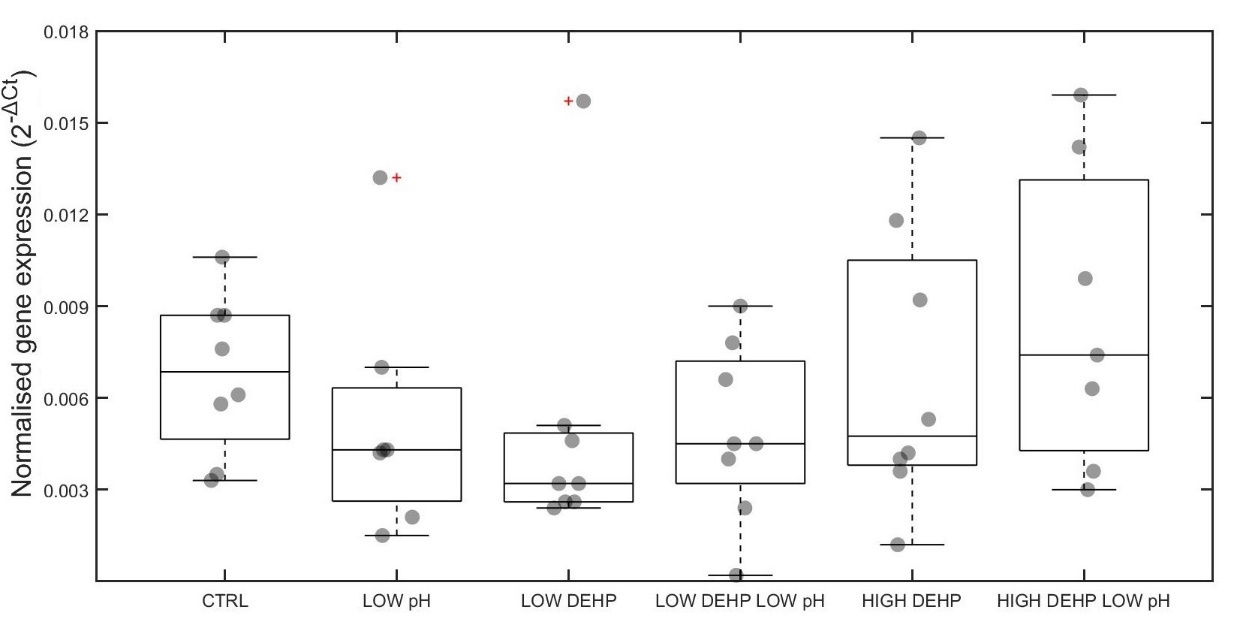


# Supplementary Fig. 5 Boxplot graphs showing *hsp70* mRNA expression in males, n= 7-8. Abbreviations are control (CTRL), low pH (LOW pH), low DEHP concentration (LOW DEHP), low DEHP at low pH (LOW DEHP LOW pH), high DEHP concentration (HIGH DEHP) and high DEHP at low pH (HIGH DEHP LOW pH). Each datapoint in grey represents the normalised gene expression as 2^-ΔCt^ values. Values are also reported in detail in Supplementary Table 4

#
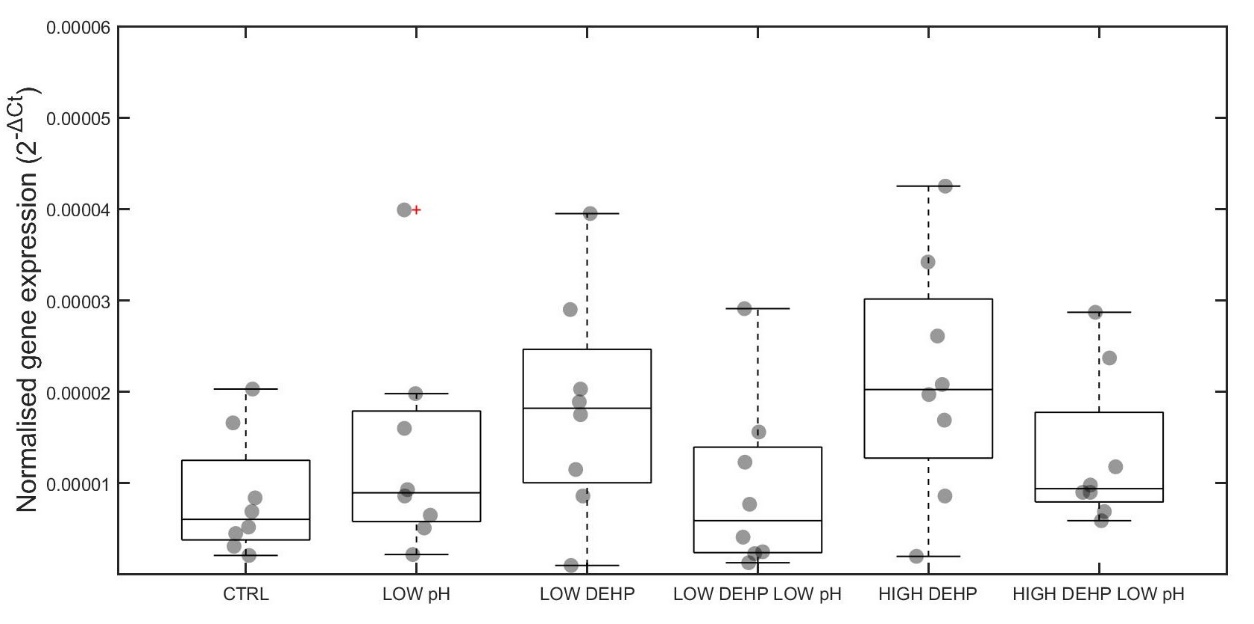


# Supplementary Fig. 6 Boxplot graphs showing *CA2* mRNA expression in males, n= 8. Abbreviations are control (CTRL), low pH (LOW pH), low DEHP concentration (LOW DEHP), low DEHP at low pH (LOW DEHP LOW pH), high DEHP concentration (HIGH DEHP) and high DEHP at low pH (HIGH DEHP LOW pH). Each datapoint in grey represents the normalised gene expression as 2^-ΔCt^ values. Values are also reported in detail in Supplementary Table 4

##
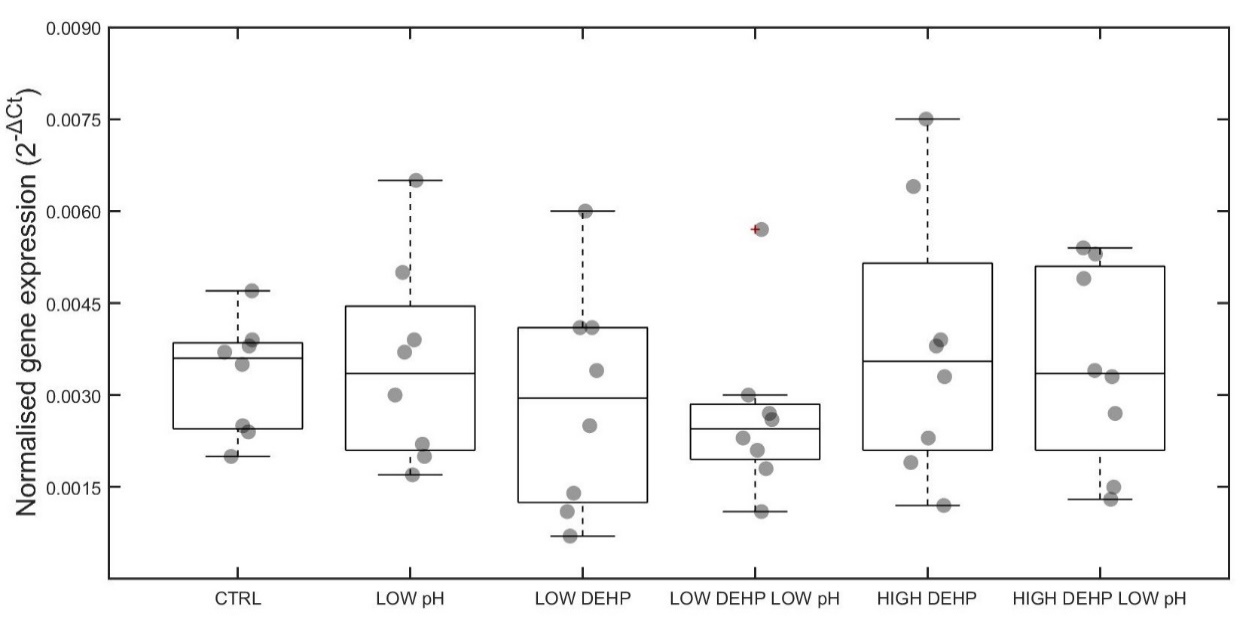


# Supplementary Fig. 7 Boxplot graphs showing *MeER1* mRNA expression in males, n= 8. Abbreviations are control (CTRL), low pH (LOW pH), low DEHP concentration (LOW DEHP), low DEHP at low pH (LOW DEHP LOW pH), high DEHP concentration (HIGH DEHP) and high DEHP at low pH (HIGH DEHP LOW pH). Each datapoint in grey represents the normalised gene expression as 2^-ΔCt^ values. Values are also reported in detail in Supplementary Table 4

##
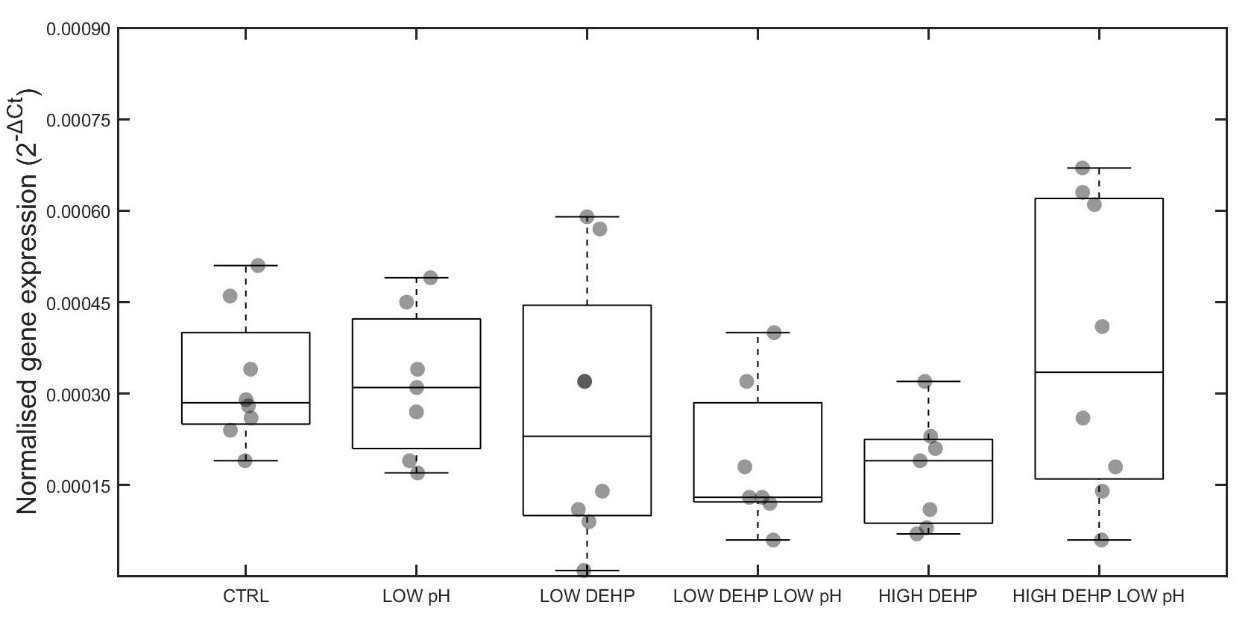


**Supplementary Fig. 8** Boxplot graphs showing *MeER2* mRNA expression in males, n= 7-8. Abbreviations are control (CTRL), low pH (LOW pH), low DEHP concentration (LOW DEHP), low DEHP at low pH (LOW DEHP LOW pH), high DEHP concentration (HIGH DEHP) and high DEHP at low pH (HIGH DEHP LOW pH). Each datapoint in grey represents the normalised gene expression as 2^-ΔCt^ values. Values are also reported in detail in Supplementary Table 4

##
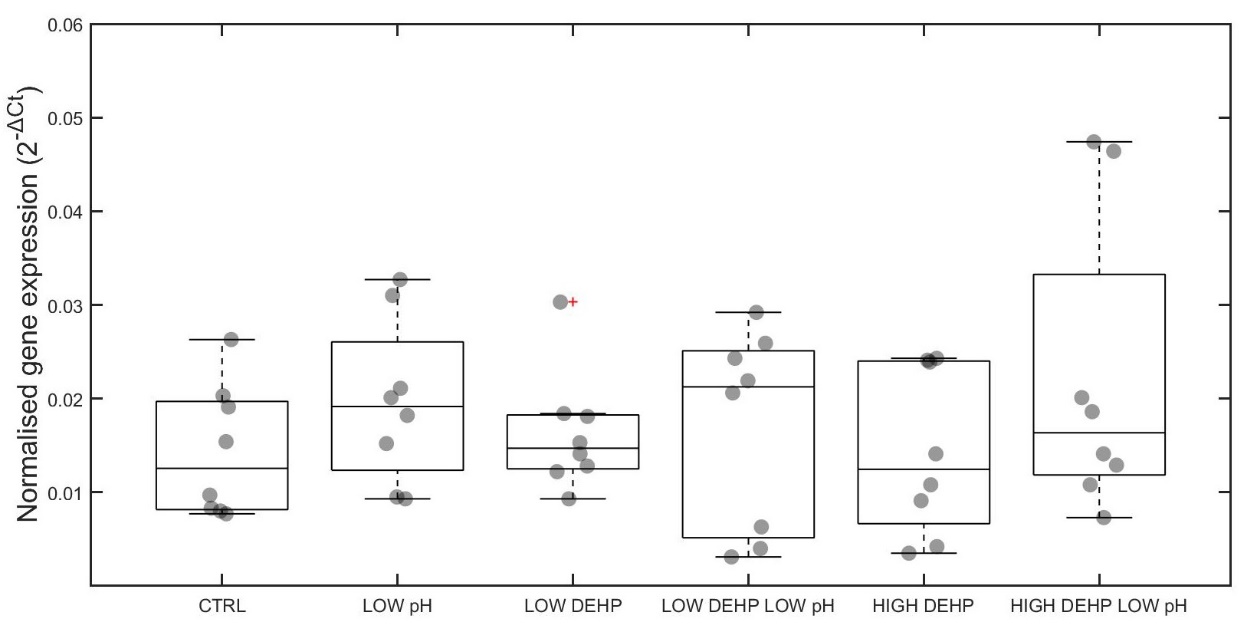


## Supplementary Fig. 9 Boxplot graphs showing *sod* mRNA expression in females, n= 8. Abbreviations are control (CTRL), low pH (LOW pH), low DEHP concentration (LOW DEHP), low DEHP at low pH (LOW DEHP LOW pH), high DEHP concentration (HIGH DEHP) and high DEHP at low pH (HIGH DEHP LOW pH). Each datapoint in grey represents the normalised gene expression as 2^-ΔCt^ values. Values are also reported in detail in Supplementary Table 5

##

##

##
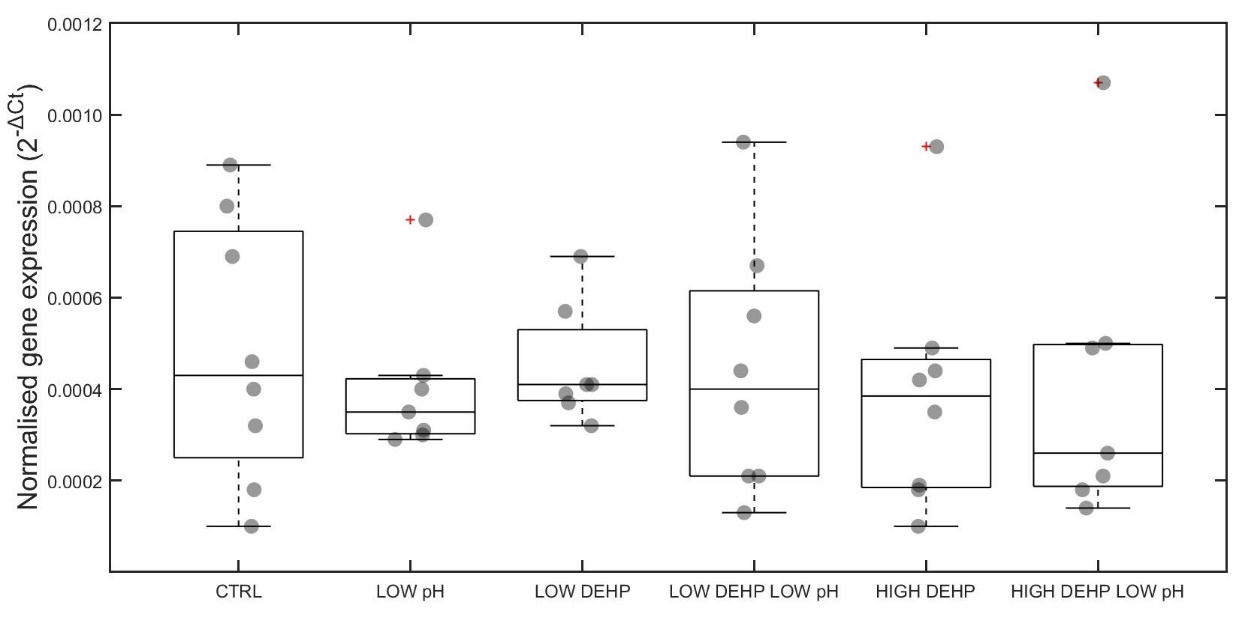


**Supplementary Fig. 10** Boxplot graphs showing *cat* mRNA expression in females, n= 7-8. Abbreviations are control (CTRL), low pH (LOW pH), low DEHP concentration (LOW DEHP), low DEHP at low pH (LOW DEHP LOW pH), high DEHP concentration (HIGH DEHP) and high DEHP at low pH (HIGH DEHP LOW pH). Each datapoint in grey represents the normalised gene expression as 2^-ΔCt^ values**.** Values are also reported in detail in Supplementary Table 5

##
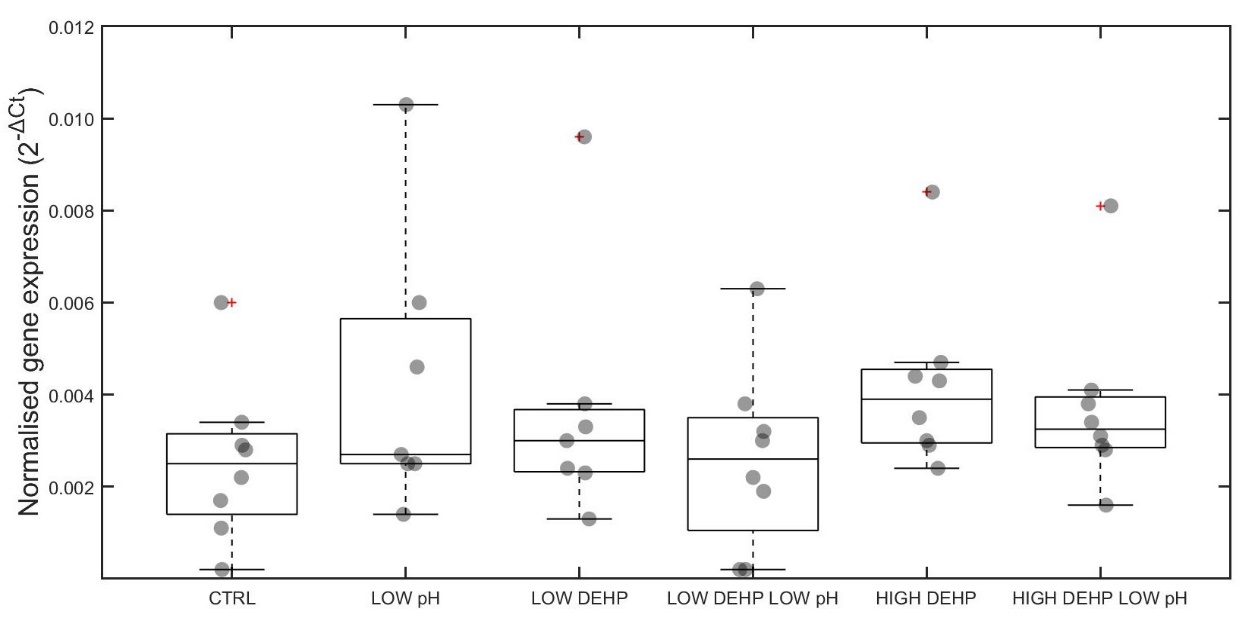


## Supplementary Fig. 11 Boxplot graphs showing *hsp70* mRNA expression in females, n= 7-8. Abbreviations are control (CTRL), low pH (LOW pH), low DEHP concentration (LOW DEHP), low DEHP at low pH (LOW DEHP LOW pH), high DEHP concentration (HIGH DEHP) and high DEHP at low pH (HIGH DEHP LOW pH). Each datapoint in grey represents the normalised gene expression as 2^-ΔCt^ values. Values are also reported in detail in Supplementary Table 5

#

##
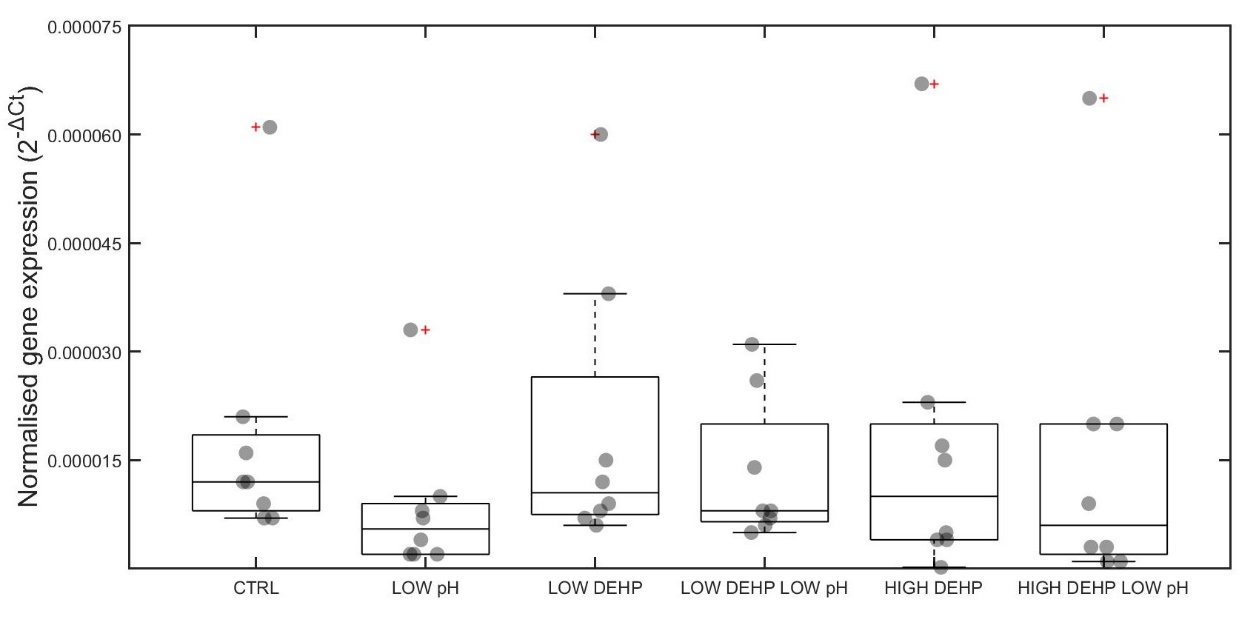


# Supplementary Fig. 12 Boxplot graphs showing *CA2* mRNA expression in females, n= 8. Abbreviations are control (CTRL), low pH (LOW pH), low DEHP concentration (LOW DEHP), low DEHP at low pH (LOW DEHP LOW pH), high DEHP concentration (HIGH DEHP) and high DEHP at low pH (HIGH DEHP LOW pH). SHR *p* value is pH = 0.09. Each datapoint in grey represents the normalised gene expression as 2^-ΔCt^ values. Values are also reported in detail in Supplementary Table 5

#
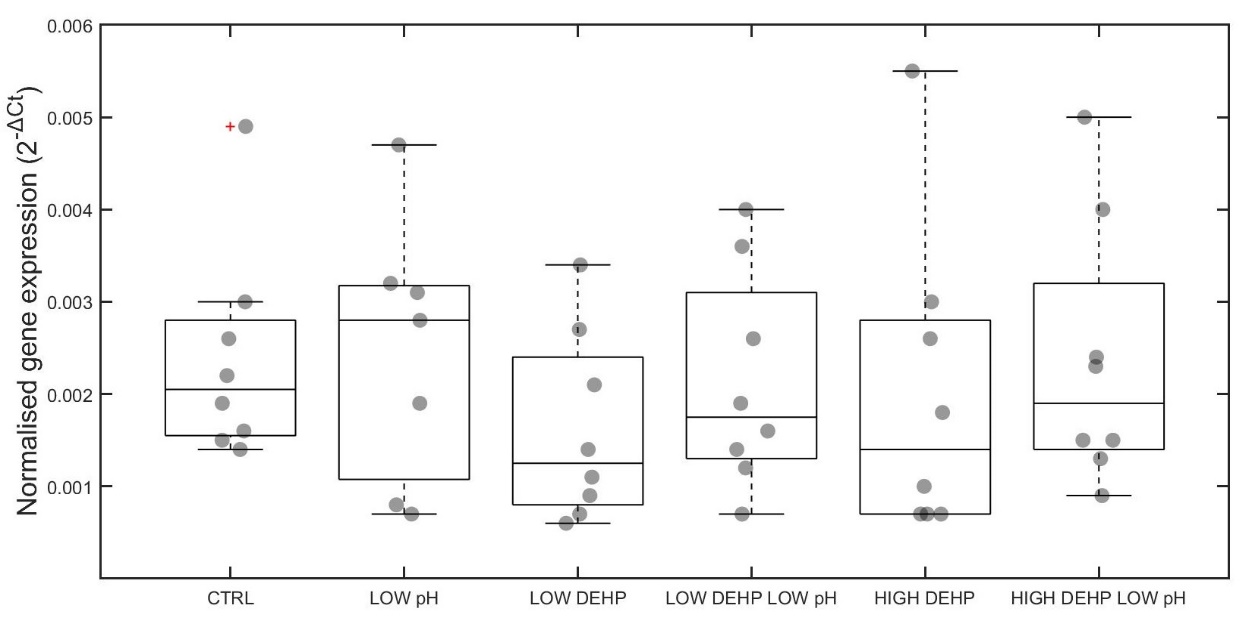


# Supplementary Fig. 13 Boxplot graphs showing *MeER1* mRNA expression in females, n= 8. Abbreviations are control (CTRL), low pH (LOW pH), low DEHP concentration (LOW DEHP), low DEHP at low pH (LOW DEHP LOW pH), high DEHP concentration (HIGH DEHP) and high DEHP at low pH (HIGH DEHP LOW pH). Each datapoint in grey represents the normalised gene expression as 2^-ΔCt^ values. Values are also reported in detail in Supplementary Table 5

##
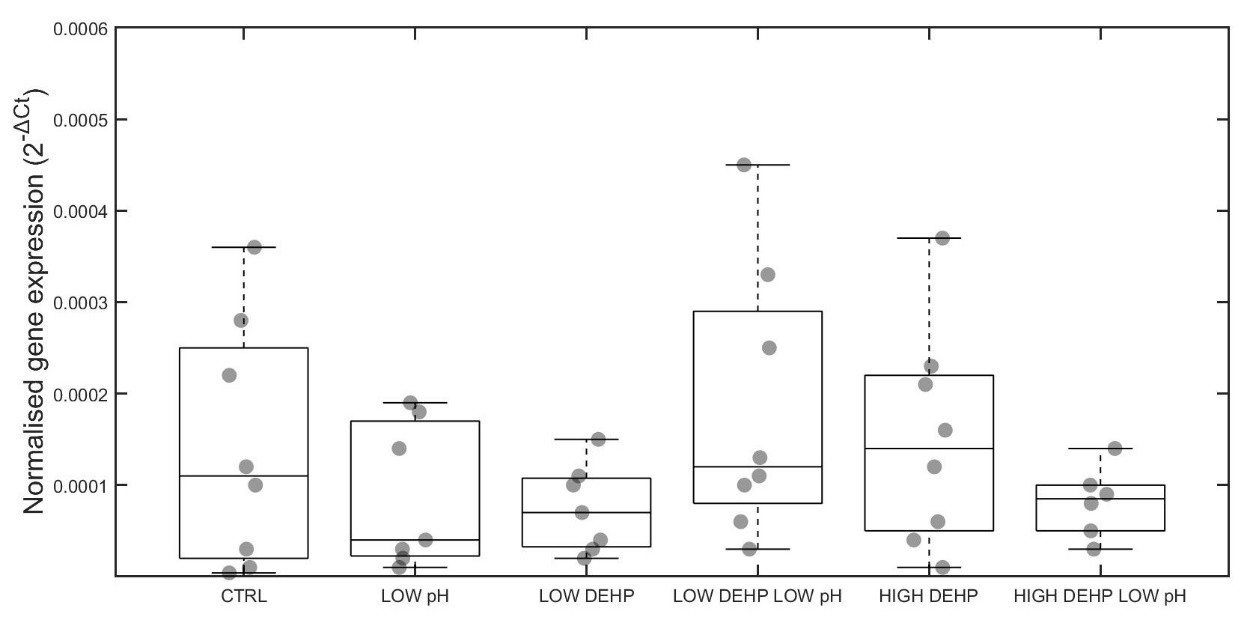


# Supplementary Fig. 14 Boxplot graphs showing *MeER2* mRNA expression in females, n= 6-8. Abbreviations are control (CTRL), low pH (LOW pH), low DEHP concentration (LOW DEHP), low DEHP at low pH (LOW DEHP LOW pH), high DEHP concentration (HIGH DEHP) and high DEHP at low pH (HIGH DEHP LOW pH). Each datapoint in grey represents the normalised gene expression as 2^-ΔCt^ values. Values are also reported in detail in Supplementary Table 5

# Supplementary Table 1 Experimental treatments and measurement of temperature, pH and alkalinity values at 35 ± 1 psu salinity. All parameters are expressed as mean ± standard deviation

| **Name of treatment** | **Description** | **Temperature**  **(°C)** | **pH**  **(Units)** | **Alkalinity**  **(mg/L)** |
| --- | --- | --- | --- | --- |
| CTRL | Control pH, no DEHP | 9.03 ± 0.34 | 8.06 ± 0.06 | 141.92 ± 9.35 |
| LOW DEHP | Low DEHP concentration | 8.76 ± 0.32 | 8.12 ± 0.09 | 136.63 ± 7.64 |
| HIGH DEHP | High DEHP concentration | 8.72 ± 0.44 | 8.12 ± 0.11 | 147.68 ± 13.59 |
| LOW pH | Low pH, no DEHP | 8.74 ± 0.36 | 7.72 ± 0.08 | 134.95 ± 9.21 |
| LOW DEHP LOW pH | Low pH and low DEHP concentration | 8.75 ± 0.34 | 7.74 ± 0.06 | 134.65 ± 14.83 |
| HIGH DEHP LOW pH | Low pH and high DEHP concentration | 8.65 ± 0.53 | 7.74 ± 0.06 | 128.48 ± 15.93 |

# Supplementary Table 2 Final primers used for qPCR amplification of reference genes and genes of interest. Sample dilutions were used in combination with primer sets in the final reactions as follows: *cat* and *CA2* (1:10), *hsp70* (1:100), *EF1α* (1:1000) and *Me18S* (1:10000)

| **Gene name** | **GenBank accession no.** | **Primer** | **Sequence (5’-3’)** | **Amplicon length (bp)** | **Amplification efficiency**  **%** | **Final conc. (nM)** |
| --- | --- | --- | --- | --- | --- | --- |
| *sod* | **AJ581746** | For  Rev | TCTCGCAGTTTACGGTCACT  GTGGAAACCGTGTTCTCCTG | 208 | 92.3 | 200 |
| *cat* | **AY580271** | For  Rev | ACTTCGACCAGAGACAACCC  GCCTGTCCATCCTTGTTGAC | 126 | 102.8 | 200 |
| *CA2* | **LK934681.1** | For  Rev | ACCAGATGGTCTTGCAGTTT  TCATCTCTGACTGCTGCTAATG | 102 | 90.2 | 200 |
| *hsp70* | **AF172607** | For  Rev | GGGTGGTGAAGACTTTGACA  TGCCCTTTCACAAGCAGTTC | 127 | 98.2 | 500 |
| *Me*  *ER1* | **AB257132** | For  Rev | CCAGATCTTCAGGGTGACGA  CTTGTTTGGCCCAGCTGATT | 94 | 95.8 | 500 |
| *Me*  *ER2* | **AB257133** | For  Rev | GGAACACAAAGAAAAGAAAGGAAG  ACAAATGTGTTCTGGATGGTG | 232 | 97.8 | 300 |
| *Me*  *28S* | **Z29550** | For  Rev | AGCCACTGCTTGCAGTTCTC  ACTCGCGCACATGTTAGACTC | 143 | 91.4 | 300 |
| *EF1* | **AF063420** | For  Rev | CACCACGAGTCTCTCCCAGA  GCTGTCACCACAGACCATTCC | 105 | 100.2 | 300 |
| *Me*  *18S* | **L33448** | For  Rev | GTGCTCTTGACTGAGTGTCTCG  CGAGGTCCTATTCCATTATTCC | 116 | 96.9 | 300 |

# Supplementary Table 3 Model classification, number of estimated parameters (K) for each model, Akaike Information Criterion (AICc), delta AIC (ΔAIC), Akaike weights (AICcWT), cumulative Akaike weights (CumWT), log-likelihood of each model (LL) for the three independent variables (+) pH (pH), DEHP concentration (DEHP) and sex (SEX) and their interactions (*) on gametogenesis stages

| **model** | **K** | **AICc** | Δ **AIC** | **AICcWT** | **Cum WT** | **LL** |
| --- | --- | --- | --- | --- | --- | --- |
| SEX | 4 | 358.08 | 0.00 | 0.820 | 0.820 | -174.92 |
| SEX + pH | 6 | 361.75 | 3.66 | 0.132 | 0.952 | -174.62 |
| SEX * pH | 8 | 364.84 | 6.76 | 0.028 | 0.980 | -173.98 |
| SEX + DEHP | 8 | 365.79 | 7.72 | 0.017 | 0.997 | -174.46 |
| SEX + DEHP + pH | 10 | 369.67 | 11.60 | 0.003 | 0.999 | -174.17 |

# Supplementary Table 4 qPCR-determined mRNA expression levels of *sod*, *cat*, *hsp70,* *CA2*, *MeER1*, *MeER2* without outliers (blank fields) expressed as 2^−ΔCt^ values in control (CTRL), low pH (LOW pH), low DEHP concentration (LOW DEHP), low DEHP at low pH (LOW DEHP LOW pH), high DEHP concentration (HIGH DEHP) and high DEHP at low pH (HIGH DEHP LOW pH in males

| **Sample no.** | ***sod***  ***(*2^−ΔCt^)** | ***cat***  ***(*2^−ΔCt^)** | ***hsp70***  ***(*2^−ΔCt^)** | ***CA2***  ***(*2^−ΔCt^)** | ***MeER1***  ***(*2^−ΔCt^)** | ***MeER2***  ***(*2^−ΔCt^)** |
| --- | --- | --- | --- | --- | --- | --- |
| CTRL 1 | 0.0071 | 0.00023 | 0.0035 | 0.0000031 | 0.0020 | 0.00019 |
| CTRL 2 | 0.0156 | 0.00031 | 0.0033 | 0.0000203 | 0.0038 | 0.00026 |
| CTRL 3 | 0.0072 | 0.00029 | 0.0087 | 0.0000045 | 0.0024 | 0.00024 |
| CTRL 4 | 0.0157 |  | 0.0076 | 0.0000069 | 0.0047 | 0.00051 |
| CTRL 5 | 0.0054 | 0.00050 | 0.0058 | 0.0000166 | 0.0025 | 0.00046 |
| CTRL 6 | 0.0042 | 0.00022 | 0.0061 | 0.0000021 | 0.0035 | 0.00034 |
| CTRL 7 | 0.0067 | 0.00036 | 0.0106 | 0.0000084 | 0.0037 | 0.00029 |
| CTRL 8 | 0.0080 | 0.00024 | 0.0087 | 0.0000052 | 0.0039 | 0.00028 |
| LOW pH 1 | 0.0113 | 0.00040 | 0.0070 | 0.0000093 | 0.0022 | 0.00034 |
| LOW pH 2 | 0.0083 | 0.00039 | 0.0043 | 0.0000160 | 0.0050 | 0.00045 |
| LOW pH 3 | 0.0095 | 0.00041 | 0.0015 | 0.0000198 | 0.0020 | 0.00019 |
| LOW pH 4 | 0.0110 | 0.00031 | 0.0021 | 0.0000051 | 0.0030 | 0.00017 |
| LOW pH 5 | 0.0081 | 0.00020 | 0.0132 | 0.0000022 | 0.0039 | 0.00031 |
| LOW pH 6 | 0.0044 | 0.00047 | 0.0043 | 0.0000086 | 0.0017 | 0.00027 |
| LOW pH 7 | 0.0099 | 0.00071 | 0.0042 | 0.0000399 | 0.0065 |  |
| LOW pH 8 | 0.0113 | 0.00017 |  | 0.0000065 | 0.0037 | 0.00049 |
| LOW DEHP 1 | 0.0058 | 0.00032 | 0.0026 | 0.0000396 | 0.0014 | 0.00032 |
| LOW DEHP 2 | 0.0112 | 0.00014 | 0.0032 | 0.0000290 | 0.0011 | 0.00014 |
| LOW DEHP 3 | 0.0035 | 0.00016 | 0.0024 | 0.0000010 | 0.0007 | 0.00001 |
| LOW DEHP 4 |  | 0.00048 | 0.0157 | 0.0000086 | 0.0060 | 0.00011 |
| LOW DEHP 5 | 0.0096 | 0.00038 | 0.0046 | 0.0000115 | 0.0034 | 0.00057 |
| LOW DEHP 6 | 0.0127 | 0.00068 | 0.0051 | 0.0000175 | 0.0041 | 0.00059 |
| LOW DEHP 7 | 0.0072 | 0.00008 | 0.0026 | 0.0000189 | 0.0025 | 0.00032 |
| LOW DEHP 8 | 0.0034 | 0.00015 | 0.0032 | 0.0000203 | 0.0041 | 0.00009 |
| LOW DEHP LOW pH 1 | 0.0029 | 0.00009 | 0.0066 | 0.0000013 | 0.0011 | 0.00013 |
| LOW DEHP LOW pH 2 | 0.0055 |  | 0.0002 | 0.0000025 | 0.0021 | 0.00012 |
| LOW DEHP LOW pH 3 | 0.0086 | 0.00021 | 0.0078 | 0.0000077 | 0.0023 | 0.00018 |
| LOW DEHP LOW pH 4 | 0.0098 | 0.00049 | 0.0040 | 0.0000123 | 0.0026 | 0.00032 |
| LOW DEHP LOW pH 5 | 0.0078 | 0.00041 | 0.0024 | 0.0000023 | 0.0018 | 0.00006 |
| LOW DEHP LOW pH 6 | 0.0073 | 0.00043 | 0.0045 | 0.0000041 | 0.0027 | 0.00040 |
| LOW DEHP LOW pH 7 | 0.0113 | 0.00045 | 0.0045 | 0.0000291 | 0.0057 |  |
| LOW DEHP LOW pH 8 | 0.0030 | 0.00007 | 0.0090 | 0.0000156 | 0.0030 | 0.00013 |
| HIGH DEHP 1 | 0.0186 | 0.00056 | 0.0118 | 0.0000208 | 0.0039 | 0.00023 |
| HIGH DEHP 2 | 0.0053 | 0.00012 | 0.0053 | 0.0000086 | 0.0012 | 0.00008 |
| HIGH DEHP 3 |  | 0.00077 | 0.0092 | 0.0000342 | 0.0038 | 0.00007 |
| HIGH DEHP 4 | 0.0055 | 0.00021 | 0.0040 | 0.0000020 | 0.0019 | 0.00021 |
| HIGH DEHP 5 | 0.0102 | 0.00083 | 0.0036 | 0.0000197 | 0.0033 | 0.00011 |
| HIGH DEHP 6 | 0.0138 | 0.00072 | 0.0145 | 0.0000425 | 0.0075 |  |
| HIGH DEHP 7 | 0.0063 | 0.00012 | 0.0042 | 0.0000261 | 0.0023 | 0.00019 |
| HIGH DEHP 8 | 0.0137 |  | 0.0012 | 0.0000169 | 0.0064 | 0.00032 |
| HIGH DEHP LOW pH 1 | 0.0108 | 0.00034 | 0.0099 | 0.0000090 | 0.0053 | 0.00063 |
| HIGH DEHP LOW pH 2 | 0.0121 | 0.00032 | 0.0142 | 0.0000098 | 0.0013 | 0.00014 |
| HIGH DEHP LOW pH 3 | 0.0076 | 0.00028 | 0.0036 | 0.0000090 | 0.0015 | 0.00006 |
| HIGH DEHP LOW pH 4 | 0.0132 | 0.00030 | 0.0074 | 0.0000118 | 0.0034 | 0.00067 |
| HIGH DEHP LOW pH 5 | 0.0179 | 0.00045 |  | 0.0000237 | 0.0054 | 0.00041 |
| HIGH DEHP LOW pH 6 | 0.0089 | 0.00032 | 0.0063 | 0.0000059 | 0.0049 | 0.00061 |
| HIGH DEHP LOW pH 7 | 0.0046 | 0.00022 | 0.0159 | 0.0000287 | 0.0033 | 0.00026 |
| HIGH DEHP LOW pH 8 | 0.0043 | 0.00019 | 0.0030 | 0.0000069 | 0.0027 | 0.00018 |

#

# Supplementary Table 5 qPCR-determined mRNA expression levels of *sod*, *cat*, *hsp70,* *CA2*, *MeER1*, *MeER2* without outliers (blank fields) expressed as 2^−ΔCt^ values in control (CTRL), low pH (LOW pH), low DEHP concentration (LOW DEHP), low DEHP at low pH (LOW DEHP LOW pH), high DEHP concentration (HIGH DEHP) and high DEHP at low pH (HIGH DEHP LOW pH in females

| **Sample no.** | ***sod***  ***(*2^−ΔCt^)** | ***cat***  ***(*2^−ΔCt^)** | ***hsp70***  ***(*2^−ΔCt^)** | ***CA2***  ***(*2^−ΔCt^)** | ***MeER1***  ***(*2^−ΔCt^)** | ***MeER2***  ***(*2^−ΔCt^)** |
| --- | --- | --- | --- | --- | --- | --- |
| CTRL 1 | 0.0077 | 0.00069 | 0.0034 | 0.000061 | 0.0049 | 0.00022 |
| CTRL 2 | 0.0203 | 0.00089 | 0.0060 | 0.000021 | 0.0030 | 0.00028 |
| CTRL 3 | 0.0083 | 0.00040 | 0.0017 | 0.000007 | 0.0015 | 0.00003 |
| CTRL 4 | 0.0263 | 0.00046 | 0.0028 | 0.000009 | 0.0019 | 0.00010 |
| CTRL 5 | 0.0154 | 0.00032 | 0.0022 | 0.000012 | 0.0014 | 0.00001 |
| CTRL 6 | 0.0097 | 0.00018 | 0.0029 | 0.000007 | 0.0022 | 0.00036 |
| CTRL 7 | 0.0080 | 0.00010 | 0.0002 | 0.000016 | 0.0016 | 0.000004 |
| CTRL 8 | 0.0191 | 0.00080 | 0.0011 | 0.000012 | 0.0026 | 0.00012 |
| LOW pH 1 | 0.0201 | 0.00029 | 0.0060 | 0.000008 | 0.0019 |  |
| LOW pH 2 | 0.0093 | 0.00077 | 0.0046 | 0.000010 | 0.0032 | 0.00004 |
| LOW pH 3 | 0.0211 | 0.00043 | 0.0027 | 0.000007 | 0.0028 | 0.00014 |
| LOW pH 4 | 0.0182 | 0.00040 |  | 0.000002 | 0.0047 | 0.00018 |
| LOW pH 5 | 0.0310 | 0.00035 | 0.0025 | 0.000002 | 0.0031 | 0.00019 |
| LOW pH 6 | 0.0152 | 0.00031 | 0.0014 | 0.000004 | 0.0008 | 0.00002 |
| LOW pH 7 | 0.0327 |  | 0.0103 | 0.000033 |  | 0.00003 |
| LOW pH 8 | 0.0095 | 0.00030 | 0.0025 | 0.000002 | 0.0007 | 0.00001 |
| LOW DEHP 1 | 0.0303 |  |  | 0.000012 | 0.0027 |  |
| LOW DEHP 2 | 0.0122 | 0.00041 | 0.0013 | 0.000009 | 0.0006 | 0.00004 |
| LOW DEHP 3 | 0.0153 | 0.00037 | 0.0033 | 0.000007 | 0.0011 | 0.00003 |
| LOW DEHP 4 | 0.0181 | 0.00069 | 0.0024 | 0.000038 | 0.0034 | 0.00010 |
| LOW DEHP 5 | 0.0093 | 0.00032 | 0.0030 | 0.000015 | 0.0009 | 0.00007 |
| LOW DEHP 6 | 0.0184 | 0.00041 | 0.0023 | 0.000060 | 0.0021 | 0.00015 |
| LOW DEHP 7 | 0.0128 | 0.00057 | 0.0038 | 0.000008 | 0.0014 | 0.00011 |
| LOW DEHP 8 | 0.0141 | 0.00039 | 0.0096 | 0.000006 | 0.0007 | 0.00002 |
| LOW DEHP LOW pH 1 | 0.0259 | 0.00044 | 0.0022 | 0.000026 | 0.0019 | 0.00010 |
| LOW DEHP LOW pH 2 | 0.0040 | 0.00021 | 0.0032 | 0.000007 | 0.0014 | 0.00011 |
| LOW DEHP LOW pH 3 | 0.0206 | 0.00036 | 0.0019 | 0.000014 | 0.0012 | 0.00003 |
| LOW DEHP LOW pH 4 | 0.0292 | 0.00067 | 0.0002 | 0.000008 | 0.0036 | 0.00025 |
| LOW DEHP LOW pH 5 | 0.0243 | 0.00094 | 0.0063 | 0.000005 | 0.0040 | 0.00045 |
| LOW DEHP LOW pH 6 | 0.0219 | 0.00056 | 0.0030 | 0.000008 | 0.0026 | 0.00033 |
| LOW DEHP LOW pH 7 | 0.0063 | 0.00021 | 0.0002 | 0.000031 | 0.0016 | 0.00013 |
| LOW DEHP LOW pH 8 | 0.0031 | 0.00013 | 0.0038 | 0.000006 | 0.0007 | 0.00006 |
| HIGH DEHP 1 | 0.0241 | 0.00049 | 0.0043 | 0.000023 | 0.0018 | 0.00023 |
| HIGH DEHP 2 | 0.0239 | 0.00035 | 0.0029 | 0.000015 | 0.0007 | 0.00006 |
| HIGH DEHP 3 | 0.0243 | 0.00042 | 0.0044 | 0.000004 | 0.0026 | 0.00016 |
| HIGH DEHP 4 | 0.0091 | 0.00093 | 0.0047 | 0.000004 | 0.0030 | 0.00021 |
| HIGH DEHP 5 | 0.0035 | 0.00019 | 0.0084 | 0.000017 | 0.0007 | 0.00012 |
| HIGH DEHP 6 | 0.0042 | 0.00018 | 0.0030 | 0.0000002 | 0.0007 | 0.00004 |
| HIGH DEHP 7 | 0.0108 | 0.00010 | 0.0024 | 0.000005 | 0.0010 | 0.00001 |
| HIGH DEHP 8 | 0.0141 | 0.00044 | 0.0035 | 0.000067 | 0.0055 | 0.00037 |
| HIGH DEHP LOW pH 1 | 0.0186 | 0.00014 | 0.0028 | 0.000001 | 0.0015 | 0.00008 |
| HIGH DEHP LOW pH 2 | 0.0141 | 0.00050 | 0.0038 | 0.000001 | 0.0024 | 0.00014 |
| HIGH DEHP LOW pH 3 | 0.0129 | 0.00026 | 0.0034 | 0.000003 | 0.0013 | 0.00010 |
| HIGH DEHP LOW pH 4 | 0.0474 |  | 0.0029 | 0.000020 | 0.0050 |  |
| HIGH DEHP LOW pH 5 | 0.0108 | 0.00021 | 0.0041 | 0.000020 | 0.0009 | 0.00003 |
| HIGH DEHP LOW pH 6 | 0.0201 | 0.00049 | 0.0031 | 0.000009 | 0.0023 | 0.00009 |
| HIGH DEHP LOW pH 7 | 0.0464 | 0.00107 | 0.0081 | 0.000065 | 0.0040 |  |
| HIGH DEHP LOW pH 8 | 0.0073 | 0.00018 | 0.0016 | 0.000003 | 0.0015 | 0.00005 |
